# Electrolyte Evolution: A Roadmap from Solvation Structure to Next-Generation Batteries

**DOI:** 10.1007/s40820-026-02119-6

**Published:** 2026-03-10

**Authors:** Chengfeng Li, Xiangyu Chen, Lingfei Zhao, Yaojie Lei, Zhuo Yang, Kunjie Zhu, Hua-Kun Liu, Shi-Xue Dou, Yun-Xiao Wang

**Affiliations:** 1 Institute of Energy Materials Science, University of Shanghai for Science and Technology, Shanghai, 200093 People’s Republic of China; 2 School of Chemistry and Physics, Queensland University of Technology, George Street, Brisbane, QLD 4000 Australia; 3 Faculty of Science, Centre for Clean Energy Technology, School of Mathematical and Physical Sciences, University of Technology Sydney, Sydney, NSW 2007 Australia; 4 Laboratory of Advanced Materials, Shanghai Key Lab of Molecular Catalysis and Innovative Materials, Fudan University, Shanghai, 200438 People’s Republic of China

**Keywords:** Electrolyte engineering, Solvation structure, High-concentration electrolytes, Localized high-concentration electrolytes, Weakly solvating electrolytes

## Abstract

Driven by global strategies for decarbonization and carbon neutrality, renewable-energy intermittency underscores the importance of large-scale electrochemical energy storage (EES). Rechargeable batteries, as the core components within EES, have long been restricted by limitations intrinsic to conventional dilute electrolytes, including narrow electrochemical stability windows, poor low-temperature performance, high flammability, and weak compatibility with high-voltage electrodes. Regulation of solvation structure in electrolytes has emerged as a key approach to overcome these bottlenecks. This review highlights five representative strategies: highly concentrated electrolytes, localized high-concentration electrolytes, weakly solvating electrolytes, hydrogen-bond regulated electrolytes, and eutectic electrolytes. These strategies have greatly advanced Li-ion, Na-ion, Zn-ion, Li–S, Li–air, and Na–S batteries. Finally, challenges ahead and opportunities in solvation-structure design are summarized to guide innovative and sustainable progress in next-generation energy storage technologies.

## Highlights

- This review elucidates how innovative electrolytes (highly concentrated electrolytes, localized high-concentration electrolytes, etc.) reshape ion–solvent interactions.
- Solvation-structure regulation is highlighted as the key to enhanced battery performance, with its recent advances summarized across diverse battery systems.
- This review outlines challenges and opportunities in solvation-structure design to guide next-generation energy storage technologies.

## Graphical abstract

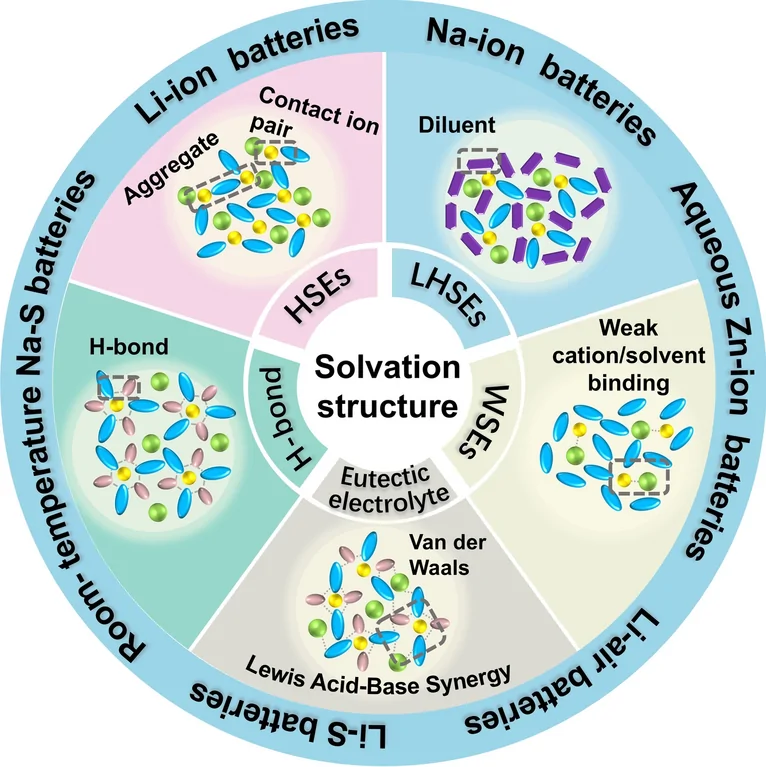

## Introduction

With the global trend of decarbonization and carbon neutralization, the full popularization of electrical equipment has become a necessary event. This has triggered a boom in research and practice toward renewable-energy sources and sustainable energy conversion/storage technologies, ranging from hydropower energy, wind energy and tides energy to solar energy. However, most of them suffer from inherent intermittency and instability. The popular strategy of using these clean energy sources for individual and industrial applications, which are known as large-scale electrochemical energy storage (EES). Among the various current technologies of EES, rechargeable batteries featured with easily portable, high energy density and energy conversion efficiency are regarded as indispensable components for portable electronic devices, electric vehicles, and energy storage stations. In particular, various battery technologies have been flourishing with multifarious advanced materials and battery chemistries of post lithium-ion batteries (LIBs) emerged since the last decades, and developed as viable choices for practical applications.

Numerous efforts, such as the high-voltage cathodes [1–5], high specific capacity anode, non-flammable electrolyte, three-dimensional current collectors, modified separator membranes, optimized binder and conductive, etc. have been widely reported and confirmed which can significantly enhance the performance of batteries. Among these studies, electrolyte engineering attracts extensive attentions due to their various useful improvement of battery performance including stable cyclability, high voltage, wide working temperature and enhanced safety, etc. Additionally, as the most widely distributed component in battery system, electrolyte is in close contact with each component in the whole battery, which play a decisive role in the performance of battery. Especially under the action of current and electric field, the electrolyte should maintain enduring chemical and physical stability to whole battery components in a variable voltage range. Thus, a key requirement for battery design is a stable electrolyte that can ensure a highly reversible electrode reaction at cathode/anode. The necessary criteria for electrolyte design are the following: (1) keep a low/no chemical-electrochemical reactivity to whole battery components; (2) a broad electrochemically stable window (ESW) can include the reversible cathodic process at a high voltage which will achieve a higher high-energy density; (3) high ionic conductivity means rapid carrier transport rate can reach a high rate performance; (4) ensure a good compatibility with electrode which can render a stable electrode/electrolyte interface (EEI). Here, EEI refers to the overall interface between the electrode and the electrolyte. This term encompasses the region of dynamic chemical and electrochemical interactions. It is commonly used to describe the compatibility and stability between the cathode or anode and the electrolyte. In contrast, the solid electrolyte interphase (SEI) specifically denotes the solid passivation layer formed on the anode surface. This layer is a thin film composed of electrolyte decomposition products. Its primary functions are to prevent further electrolyte decomposition, suppress lithium dendrite growth, and facilitate reversible Li^+^ intercalation/deintercalation. Consequently, the SEI enhances the battery’s cycle life and safety. In fact, there are many works on electrolyte engineering improved the various performance of batteries. Many well-designed combinations of salts, solvents and additives have been reported in recent years, which can be used to partially fulfill the above requirements. Such as sulfone-based solvent, nitrile-based additive, fluorine-containing additive, etc. have been confirmed that can widen ESW. But these components still suffer from some intrinsic drawbacks like highly sensitive to water, highly flammable, poor stability and so on. Other efforts, such as the synthesis of new solvents and salts also are growing popularity in the key research of batteries. However, most of them with some inevitable dilemma still in infant stage, such as lower yields, tough synthetic pathway, pricey precursor, arduous purification process and so on. The solution of all these issues requires long-term exploration and well-established process design, which is hard to be applied to the existing electrolyte in a short time.

Thus, the invention of electrolyte should not only focus on some new solvent-salt combinations. A typical liquid electrolyte contains salts in polar solvents, where ions (e.g., Li^+^, Na^+^) are solvated by surrounding solvent molecules. The geometric arrangement and chemical composition of this solvation structure are key determinants in forming the SEI, ultimately impacting battery performance. Thus, adjusting the ions solvation structure of electrolyte can lead to a series change of properties including ionic-transport properties, thermal stability, electrochemical performance and interfacial process, thereby enabling superior electrochemical performance of batteries by the superposition of these factors correspondingly. Especially for the electrolyte of post-LIBs technology, including various metal-ion batteries (Na/K/Zn/Mg ion batteries, etc.). For example, although there are many similar properties between lithium and sodium, they can even share some same battery components, such as polypropylene separator, sulfur-based cathode, hard carbon anode etc. However, it may be unsuitable to interchange the cation directly without changing other component in electrolyte due to overwhelming evidences, such as higher solubility of Na-based SEI than Li and different coordination numbers of Li^+^ and Na^+^ in same solvent etc. [6]. In addition, the different metal ions tend to experience de-solvation processes with different de-solvation energy during multiple discharge/charge procedures. These factors could contribute to the diametrically opposed outcomes to expectations, if the individual differences of ions in different solvents are not considered. Even for same metal ion in different solvents with slightly different molecular structures, the coordination structure of metal ion would be affected by steric hindrance or electrostatic potential of solvent, thereby result in a completely different passivation process in the EEI. As an illustrative example, the different intercalation/de-intercalation chemistry of Li^+^ with ethylene carbonate (EC) and propylene carbonate (PC) on graphite surface has been puzzling people for a long time. The Li^+^-intercalation/de-intercalation chemistry can occur reversibly at low potential in EC while incessant irreversible reduction in PC [7, 8]. Although the content of EC exceeded 70% in 1.0 M LiPF_6_-EC/PC electrolyte, the reason of the irreversibility of this electrolyte on graphite surface is due to more than 50% PC still in the Li^+^ solvation sheath. Only when the content of PC in Li^+^ solvation sheath is reduced to less than 50%, the electrolyte shows a highly reversibility and compatibility with graphite anodes, which indicates that there is a significant impact of characteristic of ion solvation sheath on battery performance. Hence, according to different types of electrochemical energy storage devices, the ion solvation sheath of electrolyte should be reasonably design from the perspective of nanostructure of electrolyte, which will contribute to an in-depth understanding of the interaction between electrolyte and electrode, ion transport mechanism and the electrode surface passivation.

The effect of solvation structure on battery performance much more than that. In conventional dilute electrolytes (~ 1.0 M), the cation solvation sheath is dominated by strongly solvating polar solvents with most anions excluded. This configuration results in a substantial amount of free solvent molecules in the electrolyte, which exhibit poor oxidation resistance, thereby narrowing the electrochemical window and compromising compatibility with high-voltage cathode materials. Moreover, the free solvent molecules are highly susceptible to temperature variations. They tend to arrange orderly and crystallize at low temperatures, while volatilizing and igniting readily at elevated temperatures. Several electrolytes engineering have been proposed in recent years, such as “Water-in-Salt” electrolyte (WiSE), super/high-concentrated electrolytes (HCEs), localized high-concentration electrolytes (LHCE), regulating hydrogen-bond (H-bond), weakly solvating electrolytes (WSEs) and eutectic electrolyte/deep eutectic solvents (DES) etc. The application of these strategies cover almost all kind of the battery, including Li-ion batteries, Na-ion batteries, K-ion batteries, Li–S batteries, Li-air batteries and Na–S batteries etc., and these electrolytes demonstrate unusual physicochemical and electrochemical properties (such as wide ESW, high stability, high safety, and good compatibility) which have significantly enhanced battery performance in various aspects and push the electrochemical energy storage to a new stage. Most of these outstanding performances are achieved by regulating the solvation structure of electrolyte, rather than simply create a new combination of salt and solvent. Such as the ground-breaking work by Suo et al. in 2015, the WiSE successfully expanded the ESW of aqueous electrolytes from 1.23 to 3.0 V, which brings up a series of aqueous high-voltage rechargeable batteries [9]. With the high concentration of salt in the electrolyte, all water molecules participate in the ion solvation shells, and there are almost no “free” water remainders can be found. Therefore, the anions are preferentially reduced to form a robust SEI to prevent the further decomposition of water molecules. In addition, another strategy of electrolyte design, named LHCEs, is to solve the practical application problem of HCEs, which are high cost, and high viscosity. Compared with HCEs, the solvation structure of LHCEs is composed of diluted salt-solvent clusters and non-solvating molecules (these non-solvating molecules are commonly referred to as diluents, which can disperse HCEs but retain the highly concentrated salt-solvent clusters. This will be discussed in following section). These electrolytes exhibit a board commercial prospect in the application of fire resistance, low temperature, high voltage and fast charge etc. Additionally, the other electrolyte engineering strategies are based on regulating the solvation structure of cation or the interactions between solvents, such as H-bond regulation, weak solvation and eutectic electrolyte, which can also change physicochemical and electrochemical properties of electrolyte, thus leading to the superior change in battery performance. For example, the limitations of aqueous electrolyte with the narrow ESW and high melting point of water can be changed by introducing the H-bond acceptor or donor solvent in electrolyte. The large-scale H-bond network of water will be disrupted, and the H-bond between water and additive solvent will be strengthened, thus resulting in wider ESW and reduced melting point of electrolyte.

**Fig. 1:**
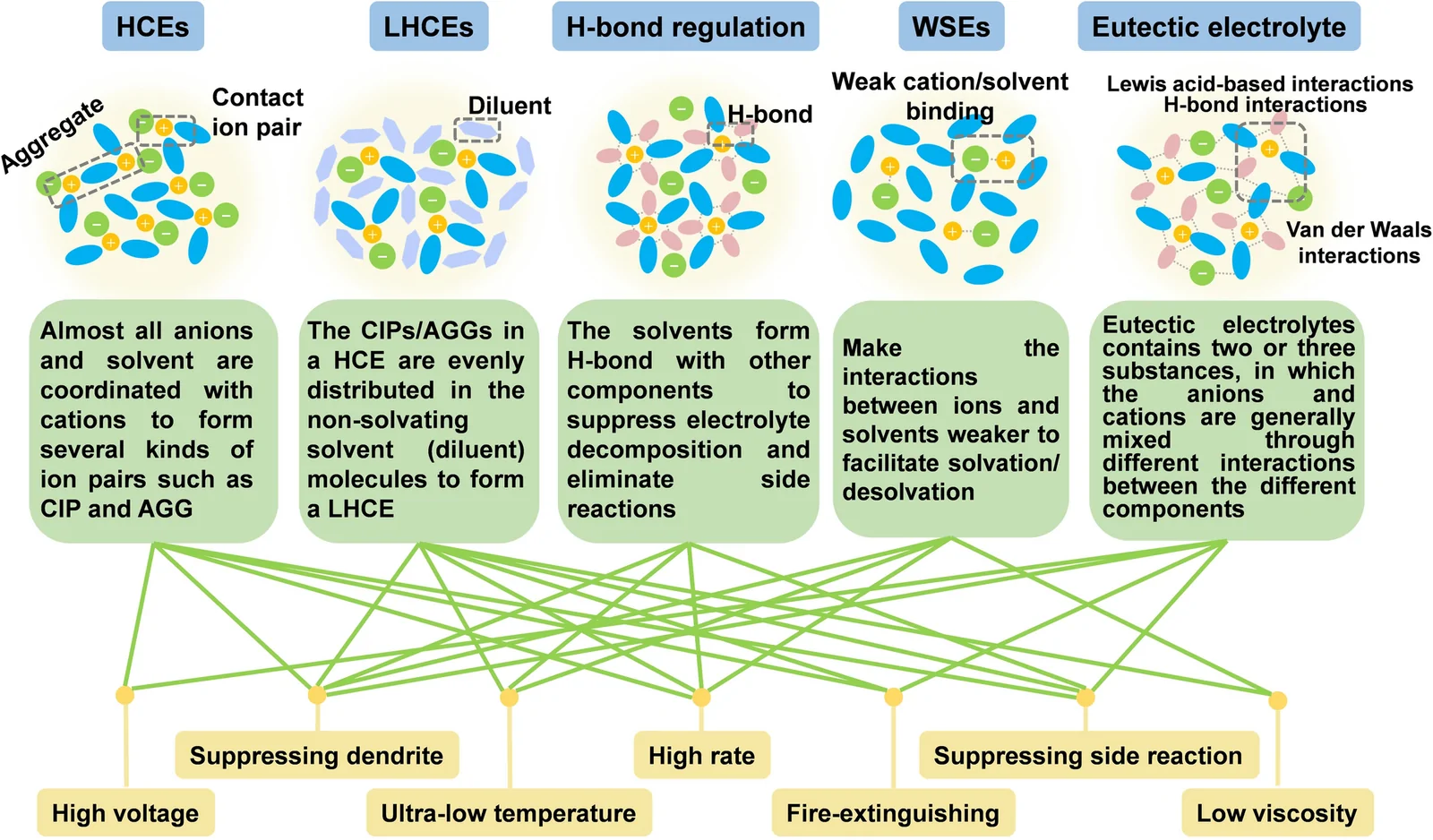
Schematic illustration of solvation-structure regulation strategies in electrolyte design, categorized into HCEs, LHCEs, hydrogen-bond regulation, WSEs, and eutectic electrolytes

These electrolyte design represent an undoubtable advantage, offering an excellent starting launch pad for the development of the innovative, sustainable and high-performance electrochemical energy storage technology. Despite the large number of review articles focusing on electrolyte design, several significant limitations remain. Most classifications are still broad and often restricted to a single battery system. They rarely offer a universal summary of strategies that are broadly applicable across different chemistries. This review addresses these limitations. It moves beyond single-system viewpoints and covers multiple electrochemical energy storage technologies, including Li/Na/Zn ion batteries, Li–S batteries, Li–air batteries, and Na–S batteries. It systematically verifies the universality of solvation-structure regulation strategies across these systems. This work also fills a critical gap in existing reviews, which lack cross-system comparisons and therefore fail to highlight the general applicability of many electrolyte design concepts. In this review, we mainly summarize these strategies for realizing various advanced battery functions by regulating the solvation structure or adjusting the interaction between solvent to solvent/additive/diluent in the electrolyte. We mainly divide these electrolyte designs into five categories based on different regulatory mechanisms including HCEs, LHCEs, H-bond regulation, WSEs and eutectic electrolyte. The corresponding solvation-structure regulation strategies are shown in Fig. 1. Then, we review recent advances in these electrolyte systems for different types of battery including aqueous battery, Li/Na/Zn ion batteries, Li–S batteries, Li–air batteries and Na–S batteries, etc. Based on reviews of the literature, the essential challenge from a perspective of electrolyte structure design is proposed, including advanced characterization techniques of solvation structure, ion transport mechanism, SEI.

## Necessity and Landscape of Electrolyte Design

### Insufficient Competence of Conventional Electrolyte

Since the advent of the commercial LIBs in 1994, EC-centric electrolytes have become the mainstream in various related field as its outstanding electrochemical performance and high compatibility with various electrode. However, with a worldwide trend toward developing high-energy density, high safety and wide-temperature renewable energies, and as the constantly innovating of electrode materials, the conventional EC-based electrolyte (~ 4.3 V vs. Li^+^/Li) has insufficient cathodic stability for some novel high-voltage electrode materials [4, 10]. During the charging/discharging process, solvent molecules are vulnerable to be attacked by these high-voltage cathode materials, which usually contained highly catalytic transition-metal oxides (such as high-Ni LiNi_0.8_Co_0.1_Mn_0.1_O_2_ (NCM-811); LiNi_0.5_Mn_1.5_O_4_ (LNMO)). This would consequently trigger a series of side effects, such as the formation of undesirable EEI, a great deal of deleterious gasses released, as a result, the capacity and cycle life of batteries decline sharply due to the separation of cathode and anode caused by gas production and the accumulation of electrolyte decomposition. Besides, the high melting point (~ 37 °C) and flammability of EC solvents almost constrains the use of batteries in some special situation such as low temperature and safety requirements. For example, commercial state-of-the-art LIBs suffer from severe capacity and energy loss below 0 °C, and will rarely be recommended for use below − 20 °C [11]. Even worse, in EC-centric electrolytes, sluggish ion diffusion in both electrolyte and electrode at low temperature cause massive metal ions gather on the EEI, which eventually leads to severe Li dendrites growing and potential safety concerns. Apart from that, these highly flammable electrolytes account for 15% by weight and 32% by volume of all components constituting commercial lithium-ion pouch cell, which poses a serious safety hazard. Under the abuse behaviors or damaged conditions (such as short circuits and flame attack), the generated heat results in a rapid increase in battery temperature and pressure, as a result, the battery ejects a lot of hot and flammable gases when the pressure reaches the threshold of a vent, and these conventional carbonate-based electrolytes would be fully burned as fuel which aggravating the destructiveness of the burned battery. This process would be much worse in large-scale applications that integrate thousands to millions of cells because thermal runaway can propagate from one cell to its neighbors [12]. Although many previous efforts have been made to develop functional solvents and additives (for example, add phosphorus or fluorine solvent for non-flammable electrolyte, add sulfone solvents for high-voltage electrolyte, etc.) to offset the mentioned deficiencies in conventional electrolytes, the price paid here is that the degradation of electrochemical performance of battery.

Overall, with novel electrode materials and new electrochemical system constantly emerging, clear limitations of conventional electrolyte are becoming apparent. Although the strategies of regulating electrolyte solvation structure superficially look like the compositional change or the adjustment of component proportion, the change in electrolyte nanostructure including ionic coordination, interaction of component, de-solvation/solvation processes etc. has a great impact on the performance of the whole batteries study. Just like strategies of doping and coating in material modification, quantitative models and characterizations for electrolyte design is similarly required.

### Importance of Solvation-Structure Design

**Fig. 2:**
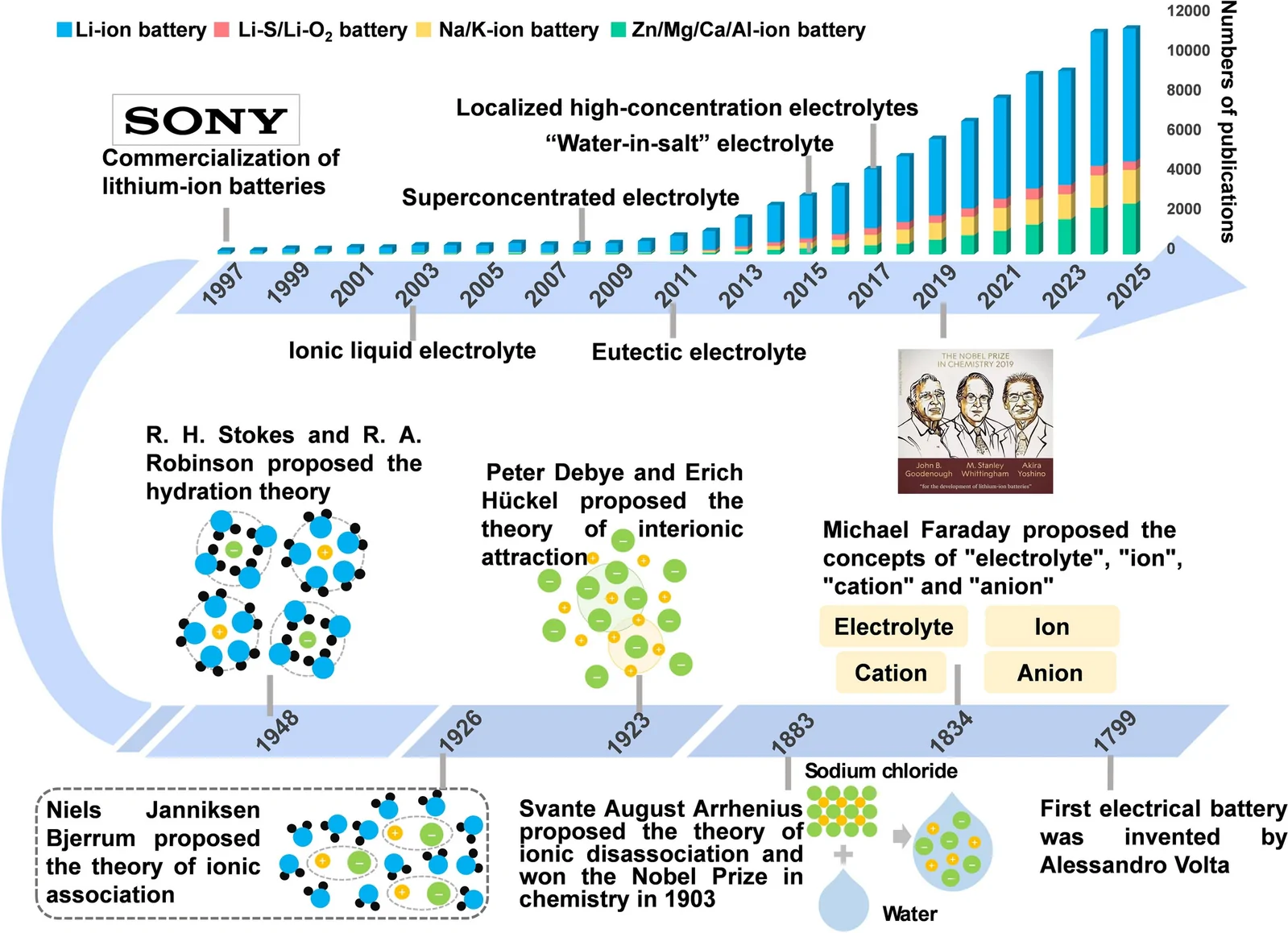
Historical timeline of electrolyte engineering and related publications since 1997

Since Michael Faraday given the name “ion”, “cation”, “anion”, and “electrolyte” in 1834, the controversy with the model of electrolyte structure has never stopped. Until 50 years later, Svante Arrhenius proposed his explanation of solid crystalline salts disassociating into paired charged particles when dissolved and won the 1903 Nobel Prize in Chemistry, the understanding of the electrolyte structure was only beginning to emerge. Especially since the first LIBs were commercialized by Sony, these solution theories, electrolyte structure and solvation model as another breakthrough of the next-generation energy storage technology has attracted extensive attention from researchers due to their significant effect on battery performance. And research activity on solvation-structure design has also surged throughout the world and set a historic high. We present a timeline of electrolyte engineering since 1997 and summarize the number of published articles on electrolyte. Placing the historical development of electrolyte could bring us in-depth understanding and inspiration into the quest for next-generation batteries (Fig. 2).

In fact, the enthusiasm of researchers for developing advanced electrode materials has never diminished, especially for LIBs, but the improvement of battery performance by electrode materials is gradually approaching the bottleneck. This dilemma highly depends on the ESW, kinetics of ion-transfer, and EEI, which can be improved to a great degree by optimizing the electrolyte chemistry and solvation structure [13, 14]. As most representative technology in recent years, HCEs and LHCEs were recognized to be the most effective methods by applying an ingenious structural design, which provide an entirely new structure at both molecular and long-range scales [15]. The HCEs effectively reduced the free solvent molecules within the cation solvation structure, leading to a lowest unoccupied molecular orbital (LUMO) shift from the solvent to the anion and primarily anion-derived SEI. In fact, cation solvation structures are fully coordinated by solvent molecules in conventional diluted electrolyte, thus the dominant species are solvent-separated ion pairs (SSIPs) and free solvent molecules. With further increase of salt concentration, the solvation structure changes gradually from SSIP in conventional electrolyte to contact ion pairs (CIPs) and aggregates in HCEs due to the reduced availability of solvent molecules. Benefit from this, the robust anion-derived EEI can be generated, and make more available options of solvent, even some solvents incompatible with the electrode can be applied to electrolyte. A typical case is that the unprecedented electrochemical stabilities to aqueous electrolytes and enabled revolutionary aqueous battery chemistries by dissolving a large amount LiFSI in water to form an aqueous HCEs of 21 M [9]. The introduction of inert diluent molecules to further dilute HCEs to form LHCEs is the compensation for the high cost and high viscosity of HCEs. This is similar to the role of chain carbonates in conventional EC-based electrolytes, but the difference is that these diluents offer little or no solubility to the salt. In the LHCEs, highly concentrated salt-solvent clusters are distributed in the non-solvating diluent molecules, and the local coordination environment of the concentrated electrolyte with no change when diluent is added. As a result, there is little effect on LUMO shifts, which indicate that the anion-derived inorganic is still the dominated component in the SEI. For example, Zhang’s group reported a BTFE-diluted LiFSI/DMC electrolyte on a LiNi_1/3_Mn_1/3_Co_1/3_O_2_ electrode enables a high Coulombic efficiency (> 99.5%) with an excellent capacity retention (> 80% after 700 cycles) [16]. The results show that the LUMO of the conduction bands are located on FSI^−^ in both the HCEs and LHCEs, which mean that FSI^−^ anions rather than DMC solvents will decompose first as the dominant reduction reaction, forming a robust FSI^−^ derived SEI layer. In general, LHCEs retains part of the characteristics of HCEs to a certain extent. And both the solvation structure of HCEs and LHCEs has significantly improved the performance of electrolyte, including the better cyclability, higher energy density, more safety, wider ESW and wider operating temperature. Other unique solvation structure, such as WSEs, have also been confirmed that can maintain liquid state in a wide-temperature range and provide a good ionic conductivity at low temperature. Additionally, the weakly solvating solution enables enhanced interaction between metal ion and anion, thereby leads to an anion-derived, inorganic-rich EEI on material surface. These abovementioned properties of WSEs ensure the normal function of batteries at low temperature, which are expected to be applied to environments with ultra-low temperature like polar expedition, space missions, etc.

Overall, the strategies of regulating electrolyte structure are not limited to abovementioned solvation structure. More effective electrolyte engineering including H-bond regulating, eutectic electrolyte, dual-ion electrolyte, co-intercalation of ion–solvent and anion regulation etc. have been confirmed that is of great significance for their performance in terms of life span, energy density, safety issues, and specific functions, and relevant mechanisms and developments these strategies will be discussed in the following sections.

## Tailoring Strategies of the Solvation Sheath

There is a commonly held belief that the influence of electrolyte on battery performance is mainly reflected in the quality of film formation on both cathode and anode. However, the formation of a stable EEI highly relies on the decomposition products derived from electrolyte. In the past few years, massive reports have validated that regulating the solvation structure of electrolyte can lead to significant changes in the component of passivation film. For example, ether solvents show relatively poor electrochemical stability against oxidation (~ 4.0 V vs. Li^+^/Li), which cannot match the high-voltage cathode. This dilemma can be altered by reducing the coordination number of solvent molecules in ionic solvation structure, consequently, ether-based electrolyte can remain stable up to more than 5.0 V without any additives. Not only that, other strategies involving regulating the coordination configuration of ionic solvation structure have also been summarized and mainly divided into five categories, including HCEs, LHCEs, regulating hydrogen-bond, WSEs and eutectic electrolyte.

### Highly Concentrated Electrolytes

#### Basic Design of HCEs

**Fig. 3:**
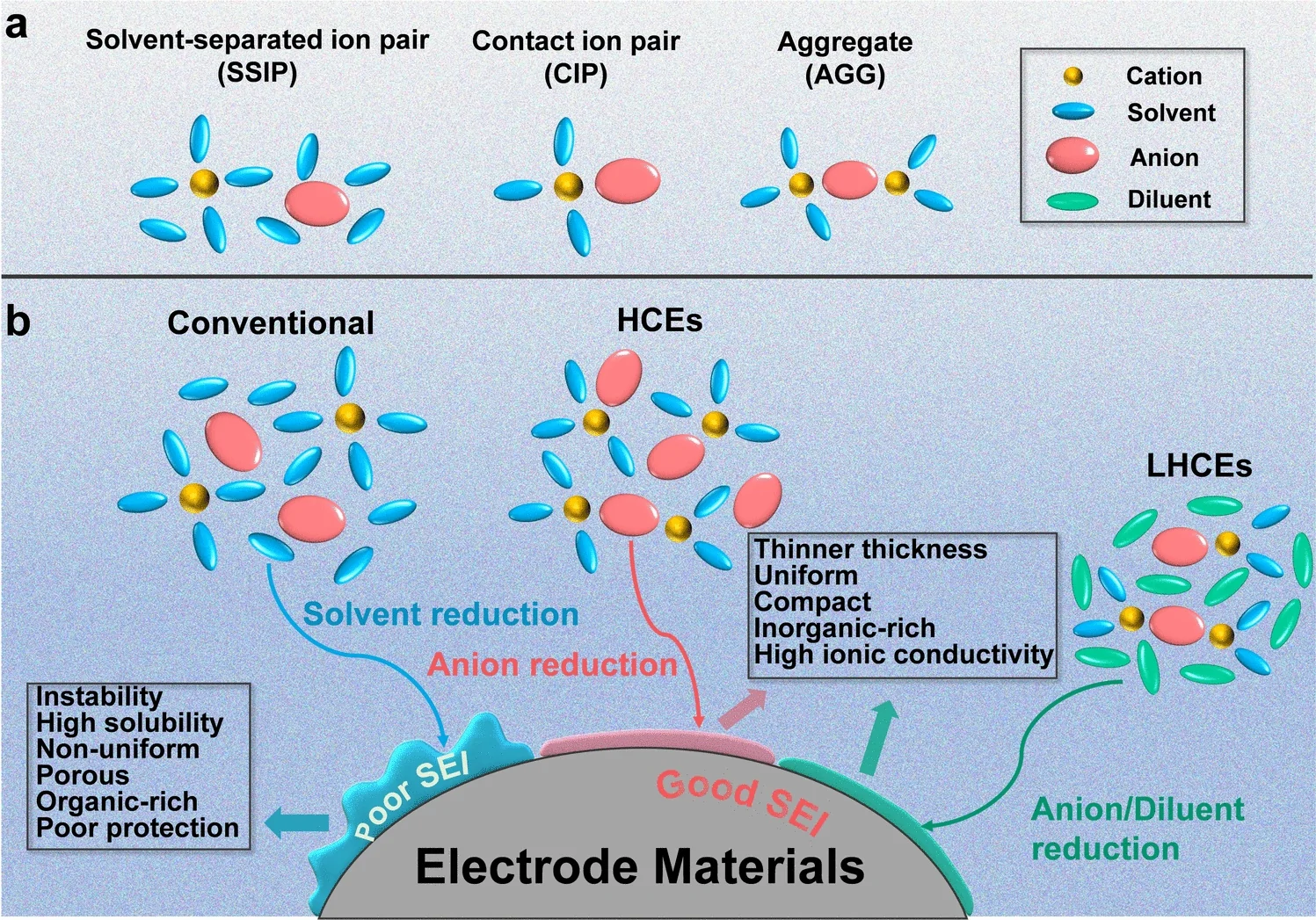
**a** Schematic illustration of several ion-solvation structures in electrolytes. **b** Comparison of the behaviors of ion-solvation structures from different electrolyte systems in constructing electrode interphase layers

Solution structures of electrolytes play dominant role in electrolyte physicochemical properties. In a salt-solvent solution, the alkali salt would exist alternated forms of salt cation solvate complexes. Owing to the different concentrations of electrolytes, the solvent molecules and anions compete to attach with alkali metal ions. As a consequence, a different ratio of solvent molecules and anions are coordinated with the alkali cations in solution. HCEs have unusual ion–solvent structures that are significantly different from those of the corresponding dilute electrolytes. As shown in Fig. 3, SSIPs (without direct interaction between anions and M cations) with stable solvating alkali cations and abundant extra free solvents coexist in dilute electrolytes. When increasing salt concentration, more solvating M^+^ molecules formed but decreasing free solvent molecules. The cations-solvent molecules show peculiar coordination structures, including CIPs (an anion coordinating to one M^+^) and aggregates (AGGs, an anion coordinating to two or more M^+^) [17–20].

**Fig. 4:**
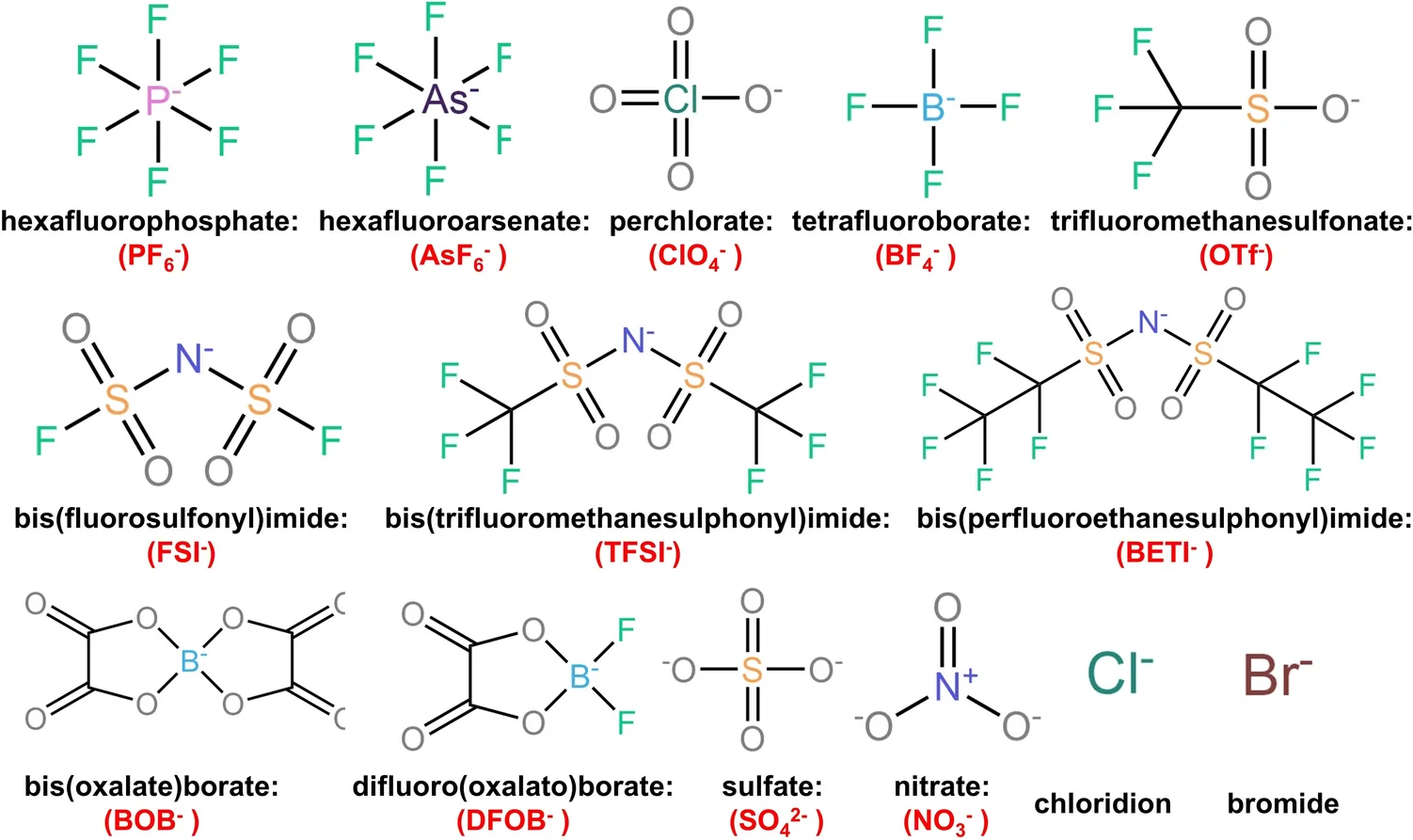
Chemical structures of representative anions used in battery electrolytes

Therefore, salts in the electrolyte consist of metal ion and complex anion group, which directly determine the electrochemical properties of the electrolyte. There are many novel salts with different anions like hexafluorophosphate (PF_6_^−^), perchlorate (ClO_4_^−^), hexafluoroarsenate (AsF_6_^−^), tetrafluoroborate (BF_4_^−^), bis(oxalato)borate (BOB^−^), difluoro(oxalato)borate (DFOB^−^), trifluoromethanesulfonate (OTf^−^), bis(fluorosulfonyl)imide (FSI^−^) bis(trifluoromethanesulfonyl)imide (TFSI^−^) and bis(perfluoroethanesulfonyl)imide (BETI^−^), etc.) have been reported and proven to be effective in the history of battery development. A schematic with the chemical structures of these and several other anions discussed in this work is presented in Fig. 4. However, the requirements of HCEs for salts need for high solubility, high dissociation constant and chemical inertness excludes most of the salts mentioned above. Until now, except for some protic solvents (such as water) with high dielectric constant and hydrogen-bond formation can dissolve most salt, only the imide-based salts (like FSI^−^, TFSI^−^ and BETI^−^, etc.) can meet the above requirements. In addition, the AsF_6_^−^-based salts with safety and toxicity concerns are inappropriate for commercial cells. ClO_4_^−^-based salts are divided into hazardous goods which is often under strict control and has certain potential safety hazards. PF_6_^−^-based salts generally exhibit a high sensitivity to hydrolysis and poor thermal stability. Thus, imide-based salts are almost the main choice of the components of HCEs. But the common issue with these anions is the Al corrosion by their electrolytes, which needs to be further optimization of salt concentration and selection of appropriate solvents in HCEs. Other salts such as Cl^−^-, Br^−^-, NO_3_^−^-, and SO_4_^2−^-based salts are often used in aqueous HCEs. This kind of salts stronger cation–anion interactions and can only be dissolved in large amounts in water or other protic solvents. But these same issues inorganic salts are used as additives rather than as main salts, due to the serious side reactions between anions with metal electrode and the formation of poor EEI.

**Fig. 5:**
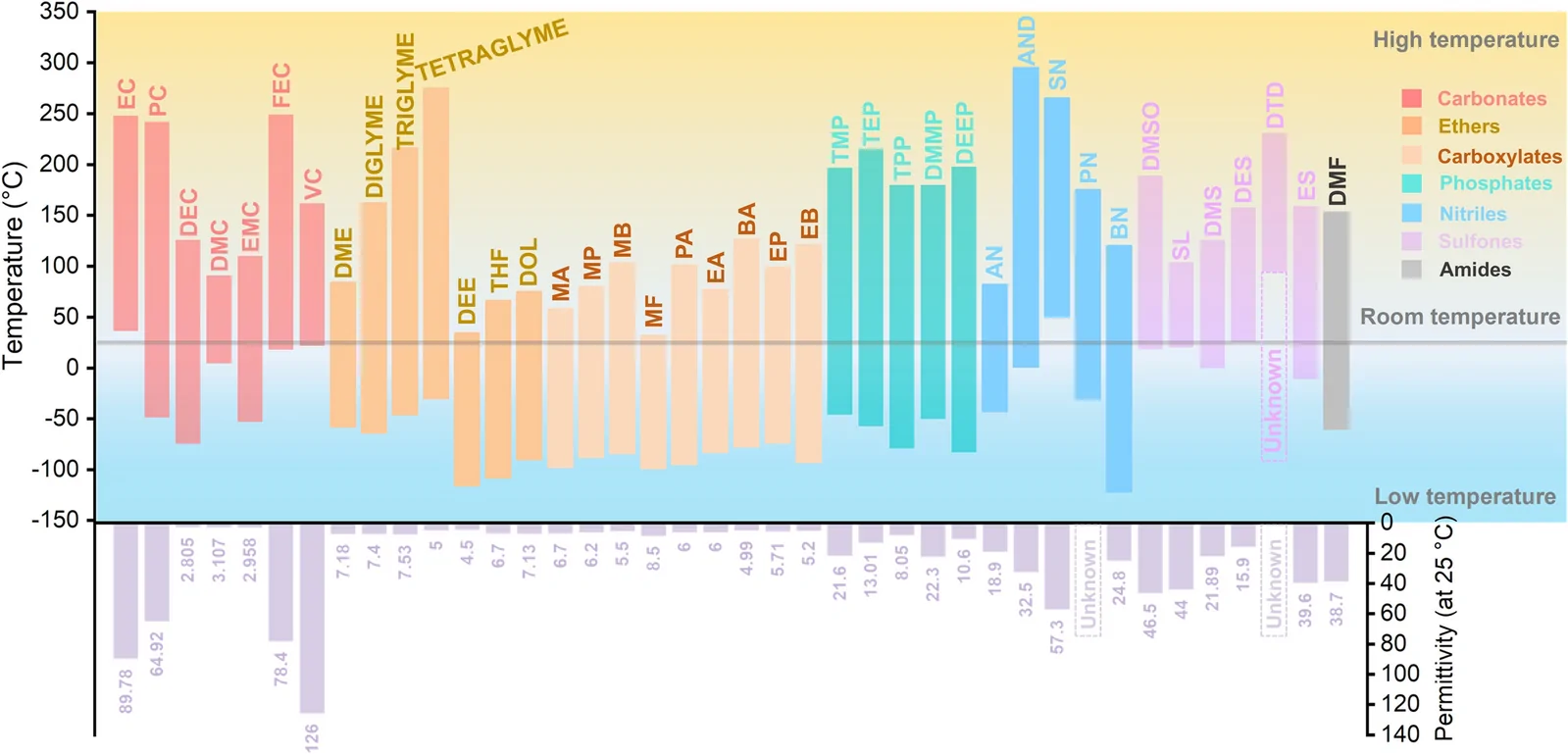
Physical properties of representative solvents for HCEs

As another part of electrolyte, the option of solution components is of no less importance for the battery performance than the option of the salt. In fact, solvating solvents have more available choice than salt, even these solvents which with inferior chemical stability toward metal (Li, Na, K, etc.). In the HCEs, the enhanced interactions between cations and anions/solvents as well as a decrease in the content of free-state solvent molecules. As a result, these salt anions participate in the EEI layer formation by shifting from a solvent decomposition to a salt anion decomposition/reaction. In recent years, massive researches have successfully applied HCEs strategy to different electrodes in various solvents that are usually considered incapable to produce effective EEI layers on its surfaces, including carbonate-based, ether-based, carboxylate-based, phosphate-based, nitrile-based, sulfone-based and amide-based. The physical properties of these solvents are shown in Fig. 5. Among various options, ether-based solvents have emerged as the predominant choice for formulating HCEs, owing to their favorable salt solubility and robust chemical stability against alkali metals. In contrast, carboxylate-based, despite their widespread use, suffer from high viscosity that significantly compromises rate capability. While phosphate-based solvents offer exceptional flame retardancy and thus improve safety, their poor compatibility with anodes limits their adoption. Other candidates, such as nitrile-based, sulfone-based and amide-based solvents, are also hindered by intrinsic shortcomings that impede overall performance. Although the unique anion-rich solvation chemistry in HCEs can partially mitigate these issues, achieving an economically viable and universally high-performing electrolyte design remains a considerable challenge. Despite these challenges, combining different solvents offers a route to overcome their individual shortcomings. An illustrative case is the formulation of HCEs using blends of high ionic conductivity nitrile-based solvents and passivation-effective carboxylate-based solvents. Precise optimization of their ratio can fully harness the unique anion-involved interfacial chemistry of the HCEs strategy, thereby pushing battery performance to a higher level. Similarly, the HCEs strategy should evolve toward multicomponent formulations. The primary solvent must be rationally designed to meet specific battery performance requirements, while cosolvents and additives demand systematic exploration of their optimal ratios and chemical compositions to unlock full functional potential.

#### Wide Electrochemically Etable Window

**Fig. 6:**
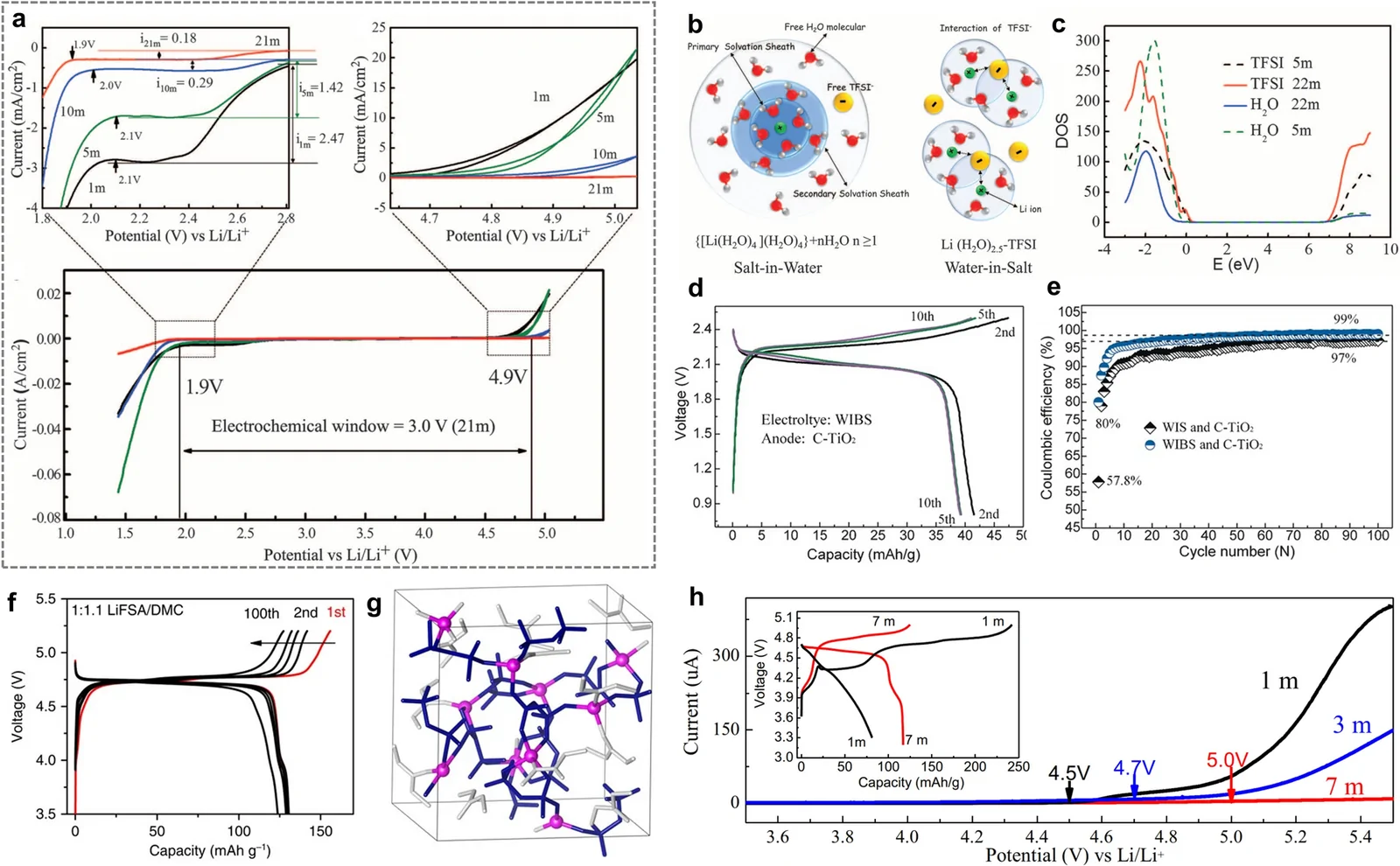
**a** Electrochemical stability window of LiTFSI-H_2_O electrolytes. **b** Illustration of the evolution of the Li^+^ primary solvation sheath in diluted and water-in-salt solutions. **c** Projected density of states (DOS) for H_2_O-LiTFSI electrolyte. **a–c** Reproduced with permission [9]. Copyright 2015 AAAS. **d** Charge–discharge profiles of the electrochemical couple of LiMn_2_O_4_/C-TiO_2_ in WIBS at the 2nd, 5th, and 100th cycles. **e** Cycling stability and Coulombic efficiencies for varying combinations of TiO_2_ anode and electrolyte. **d,** **e** Reproduced with permission [21]. Copyright 2016 Wiley-VCH. **f** Charge–discharge voltage curves of LiNi_0.5_Mn_1.5_O_4_/lithium metal half-cells using superconcentrated 1:1.1 LiFSA/DMC electrolytes at a C/5 rate. **g** Snapshots of typical equilibrium trajectories obtained by DFT-MD simulations: superconcentrated solution (10 LiFSA/11 DMC, ca. 5.5 mol dm^−3^). **f,** **g** Reproduced with permission [24]. Copyright 2016 Springer Nature. **h** Concentration-dependent oxidation potential by LSV in three-electrode device. **h** Reproduced with permission [25]. Copyright 2018, National Academy of Sciences

With the increase of the ratios of salt-to-solvent, the solvent molecules can be mostly coordinated with the cations which minimizes the presence of free solvent molecules in the electrolyte. As a result, the highest occupied molecular orbital (HOMO) of electrolyte shows a downward shift from solvent molecules to anions, and the solvent molecules exhibit higher oxidative stability in coordinated state with cations than in its free state. Benefit from this, great progress has been made toward aqueous rechargeable metal-ion batteries, which are limited by the relatively narrow ESW of the conventional aqueous electrolyte. The most extreme case mentioned above is to dissolve LiTFSI in water to reach a concentration of 21 M (or ~ 5 M), which successfully expanded the ESW of aqueous electrolytes from 1.23 to > 3 V (Fig. 6a) [9]. With LiTFSI concentrations increased to 21 M, average two TFSI^−^ and two point five H_2_O molecules would be observed in each Li^+^ primary solvation sheath, and such a high concentration of TFSI^−^ leads to premature TFSI^−^ reduction and delayed water oxidation (Fig. 6b, c). In their follow-on work, the Li^+^ concentration of electrolyte was further improved to 28 M by adding 21 M lithium bis(trifluoromethane)sulfonyl imide (LiTFSI) + 7 M lithium trifluoromethane sulfolate (LiOTF) in water [21]. Compared with single salt system (21 M LiTFSI in water), the prepared electrolyte can achieve wider ESW (from 1.83 to 4.9 V vs. Li^+^/Li) with excellent cycling stability and high coulombic efficiency. The mixed salts system broken through the limit of maximum solubility of LiTFSI in water and further increased the concentration of Li^+^ from 21 to 28 M, which effectively decreased the free water molecules and formed protective SEI on anode. As a result, the aqueous Li-ion cell which consist of LiMn_2_O_4_ cathode and carbon-coated TiO_2_ anode can deliver a high voltage of 2.5 V with energy density of 100 Wh kg^−1^ (Fig. 6d, e) [21]. Inspired by the high-concentrated aqueous Li-ion batteries, this concept has been successfully transferred to other types of aqueous electrochemical energy storage devices, including Na/K-ion batteries. Jin et al. reported a low-cost high-energy aqueous sodium-ion batteries by dissolving 17 M NaClO_4_ + 2 M NaOTF in water [22]. This high-concentrated aqueous electrolyte delivered a wide ESW of 2.8 V and enables a 1.75 V Na_3_V_2_(PO_4_)_3_||Na_3_V_2_(PO_4_)_3_ full cell to deliver an energy density of 70 Wh kg^−1^ at 1 C (with a capacity retention of 87.5% after 100 cycles). Jiang et al. showed that an aqueous K-ion batteries can achieve 70% capacity retention at 100 C and a lifespan of over 10,000 cycles by using a 22 M KCF_3_SO_3_ aqueous electrolyte with the ESW of 3.0 V [23]. Similar phenomenon was also observed in aqueous Zn ion batteries (ZIBs), however, despite Zn^2+^ concentration was increased to 30 M, the high affinity between metal cation with H_2_O molecules can inhabit the hydrogen evolution reaction (HER) and lead to wider ESW to match more electrode materials, there was still a minor presence of [Zn(H_2_O)_6_]^2+^ with six free water molecule due to the strong Coulombic interaction between Zn^2+^ and H_2_O. Consequently, the active H_2_O molecules derived from solvated Zn^2+^ are easy to be decomposed into H^+^ and OH^−^.

Organic solvents show wider commercialization value because of its high designability and buildability. Even some harmful solvents are known for serious electrode exfoliation or continuous electrolyte decomposition, which can be directly used in the battery system without any protective additive by using the strategy of HCEs and exhibit an excellent cyclability. It is well known that the irreversible intercalation/de-intercalation between Li^+^ and graphite will occur in the electrolyte which contains high amounts of PC molecules. This is because of the ceaseless irreversible reduction of PC-based electrolyte at ∼0.7 V and the consequent intensive exfoliation of the graphite layers [26]. However, this can be effectively prevented by increasing the number of Li^+^ to maximize the capture of free PC molecules [27]. Additionally, the ESW of PC-based electrolyte can be unexpectedly increased from 3.9 V at low concentration to close 5 V at high concentration, which indicate that the oxidative decomposition of electrolyte was effectively suppressed [28]. Wang et al. achieved a 5 V-class LiNi_0.5_Mn_1.5_O_4_ (LNMO)||graphite full cell by using a super-concentrated LiFSI in DMC electrolyte (Fig. 6f), which are composed of LiFSI and DMC in a molar ratio of about 1:1.1 [24]. As shown in Fig. 6g, each FSI^−^ coordinates to 2–3 Li^+^ and each Li^+^ is coordinated by 2–3 FSI^−^ in the super-concentrated LiFSI/DMC electrolyte. Consequently, each FSI^−^ connect with each Li^+^ other via the intensive association, leading to a reinforced three-dimensional network. This mechanism was further proven by using FEC solvent [25]. Benefit from the high LiF content on the cathode surface that generated by the decomposition of 7 M LFSI/FEC electrolyte, this electrolyte can satisfy simultaneously with wide ESW, good compatibility with LNMO electrode (Fig. 6h), Al curnode collector corrosion resistance, and superior reversibility of Li metal anode. More than that, similar mechanisms have also been transferred to other types of solvent. Li et al. reported a 6 V high-voltage K-based dual-graphite battery by using potassium bis(fluorosulfonyl)imide (KFSI) into tetramethylene sulfone (TMS) [29]. Sulfone-based solvents itself have the strong electron-withdrawing sulfonyl group serves to lower the energy level of the HOMO, leading to higher oxidation stability. When combined with HCEs strategy, the 5.2 M KFSI/TMS electrolyte exhibit a surprising oxidation potential of more than 6 V.

An innovative feature of HCEs is the excellent ESW, which not only supports the operation of various anodes, such as traditional graphite and silicon anode, but also enables the reversible operation of high-voltage cathodes, such as 5 V-class [24]. Dai et al. compared the electrochemical stability of commercial electrolyte (Com: 1 M LiPF_6_ in EC/DMC with volume ratio of 3/7), 1 M LiPF_6_ in dimethyl carbonate (DMC)/fluoroethylene carbonate (FEC)/1,1,1,3,3,3-hexagluoroisopropyl methyl ether (HFPM), and 3 M DMC/FEC/HFPM [25]. The linear sweep voltammetry (LSV) results indicated that the electrolytes commence to decompose when the potential values exceed 4.4, 4.7, and 5.5 V for the Com, 1 M and 3 M electrolyte, respectively. They believed that the 3 M electrolyte has fewer free solvent molecules but presents strong solvation of DMC with Li ions. As a result, the oxidation resistance of electrolyte is much enhanced. The new solution structure alters the LUMO locations from solvent toward the salt anions, which results in the reductive decomposition of the salt at high potential before the solvent. Both LNMO and LLRO can show enhanced performance, which is ascribed to the favorable LiF-rich cathode-electrolyte interface resulted from the reduction of PF_6_^−^ anions but not the solvent molecules decomposition.

#### Excellent EEI Stability

**Fig. 7:**
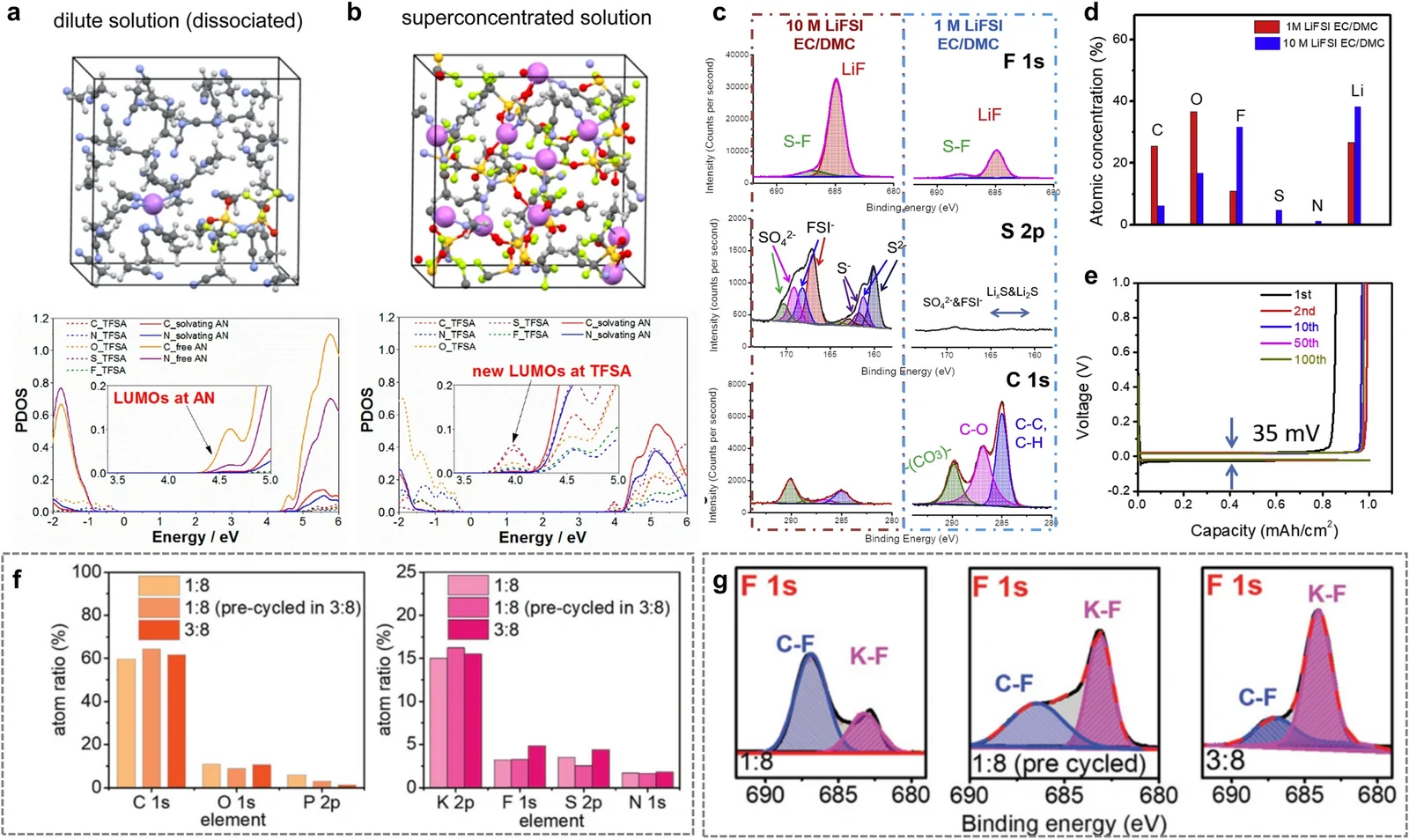
Supercells used and projected density of states (PDOS) obtained in quantum mechanical DFT-MD simulations on **a** dilute (1-LiTFSA/43-AN corresponding to 0.4 mol dm^–3^) and **b** superconcentrated (10-LiTFSA/20-AN corresponding to 4.2 mol dm^−3^) LiTFSA/AN solutions. **a,** **b** Reproduced with permission [19]. Copyright 2014, American Chemical Society. **c** XPS analysis of the SEI layers for the Li-metal anode cycled in concentrated EC/DMC and diluted EC/DMC electrolyte. F 1*s*, S 2*p*, and C 1*s* spectra are presented, including peak deconvolution and assignments. **d** Elemental concentration of the SEI layer obtained in the XPS analysis for the two Li-metal anodes. **e** Voltage profiles for the cell cycled in 10 M LiFSI-DMC. **c–e** Reproduced with permission [31]. Copyright 2018, Cell Press. **f** XPS elemental proportion analysis of graphite electrode and pre-cycled graphite electrode after being cycled in 1:8 and 3:8 electrolyte. **g** XPS fitting curves of the graphite surfaces after cycling in 1:8 and 3:8 electrolytes. **f,** **g** Reproduced with permission [32]. Copyright 2021, Wiley-VCH

One of the most striking features of HCEs is unusual stability toward electrodes, especially for anode. Some representative cases such as graphite electrode suffer from solvent co-intercalation and structure exfoliation in PC-centric electrolyte at low salt concentration, and hard carbon cannot be passivated well in flame retardant phosphate electrolyte at low salt concentration. Both of them need a SEI-forming solvent to allow their reversible cation intercalation reaction. However, just increasing the salt concentration can enable these carbonaceous anodes delivery stable reversible capacity and effectively suppress side reactions without any SEI-forming additive. These merits can be attributed to the anion-derived SEI caused by unique solvation structure of HCEs. This is because the LUMO shifts from the solvent to the anion, which is easier to receive an electron under a reductive atmosphere. Figure 7a, b shows the compassion of projected density of states (PDOS) between diluent and high-concentrated LiTFSI/AN electrolyte [19]. With increasing the concentration of LiTFSI from 1 to 4.2 M, a significant downward shift of the TFSI^−^ orbital level can be observed in PDOS profile. Beyond that, in the same electrolyte, Sodeyama et al. showed that TFSI^−^ tend to receive an excess electron to form solvation structure of AGG state, in which the each TFSI^−^ is coordinated with two or more Li^+^ cations to form a typical network chain [30]. Due to the formation of network chained structure, TFSI^−^ sacrificially accepts a reductive electron and dissociates the CF_3_ moiety. Apart from the confirmation of density functional theory-based molecular dynamics (DFT-MD) simulations, the surface analysis of X-ray photoelectron spectroscopy (XPS) also agreed with these results. For example, Fan et al. reported a high-voltage Li-metal batteries using 10 M LiFSI-EC/DMC to form highly fluorinated interphase [31]. As shown in Fig. 7c, abundant F and S can be observed in the Li-metal anode that cycled in concentrated EC/DMC electrolyte, which are all derived from FSI^−^ anions. Only trace amounts of carbon compounds were detected in the SEI film, indicating that SEI is predominantly come from the decomposition of LiFSI because C are only from the EC/DMC solvent (Fig. 7d). As a result, the F-rich interphases ensure a high Li plating/stripping Coulombic efficiency of ~ 99.3% with small polarization (Fig. 7e). Similar observations are also reported in K ion batteries by Liu et al. [32]. The graphite electrode cycling in KFSI/TMP with molar ratio of 3:8 showed the highest content of fluorine, which is a characteristic element of the FSI^−^ (Fig. 7f). Similar observations are also reported for LiTFSI/Dimethyl sulfoxide (DMSO) [33], LiFSI/TMP, and NaFSI/TMP electrolytes (Fig. 7g) [12]. All the above results suggest that the SEI is predominantly reduced by the salt anions on the surface of anode in concentrated electrolytes, which is attributed to their unusual electronic states caused by the change of electrolyte structure [34]. The anion-derived SEI exhibit not only higher stability but lower interfacial resistance compared with the solvent-derived SEI, which is beneficial to the electrochemical performance [19].

**Fig. 8:**
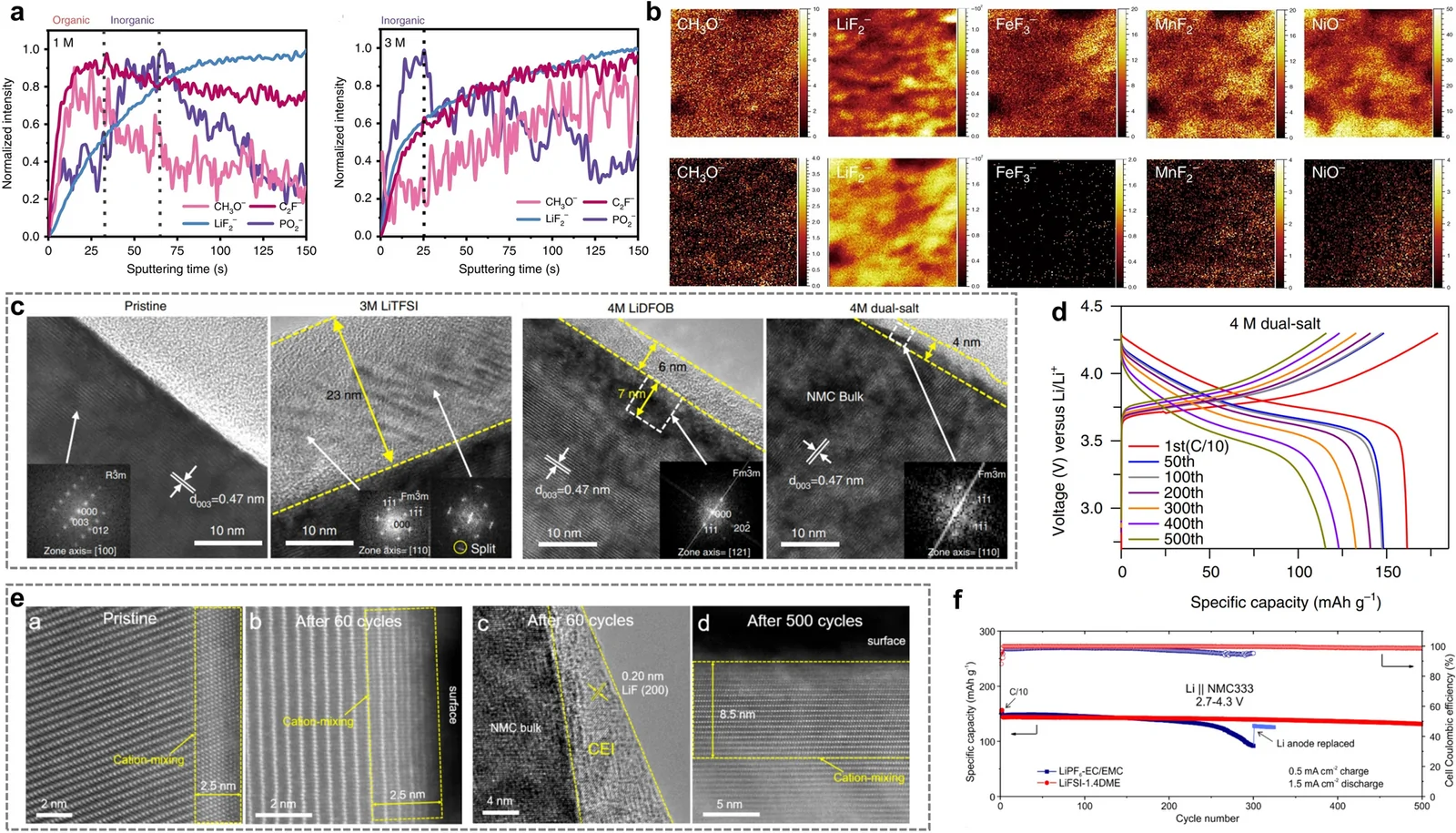
**a** TOF-SIMS spectra of interphases on the surface of cycled electrodes, Normalized (to maximum) depth profiling of several typical second ion fragments on the cycled electrode outside surface. **b** TOF-SIMS chemical maps of several typical second ion fragments with 150 s of sputtering on electrodes cycled in 1 and 3 M electrolyte, respectively. **a,** **b** Reproduced with permission [35]. Copyright 2020, Springer Nature. **c** Pristine NMC cathode and the cycled NMC cathodes (50 cycles) in different ether electrolytes: 3 M LiTFSI, 4 M LiDFOB and 4 M dual-salt. Insets are the fast Fourier transform (FFT) patterns of selected regions. **d** Voltage profiles of Li||NMC cells at selected cycles during cycling in different highly concentrated DME electrolytes. **c,** **d** Reproduced with permission [36]. Copyright 2018, Springer Nature. **e** STEM image of the pristine NMC333 sample. **f** Comparison of cycling performance of the NMC333 in the LiPF_6_–EC/EMC electrolyte (1 M LiPF_6_in EC-EMC, 3:7 by wt.) and the LiFSI-1.4DME electrolyte under 4.3 V charge cutoff voltage. **e**, **f** Reproduced with permission [37]. Copyright 2019 American Chemical Society

In fact, the anion-derived passivation layers can also be observed on the cathode surface. Liu et al. reported a concentrated 3 M LiPF_6_-EC/EMC/DMC electrolyte, which can effectively inhibit the dissolution of transition metals and stabilize the Li_1.2_Ni_0.15_Fe_0.1_Mn_0.55_O_2_ (LNFMO) cathode structure [35]. As shown in Fig. 8a, b, the surface chemical composition of LNFMO cathode was characterized by time-of-flight secondary-ion mass spectrometry (TOF–SIMS), which shows the earlier signals of LiF_2_^−^ and PO_2_^−^ than C_2_F^−^ and CH_3_O^−^ in high-concentrated electrolyte but later in diluent electrolyte. In addition, the intensity of organic species, dissolution products (MnF_x_^−^ and FeF_x_^−^), and NiO^−^ on the electrode surface are higher in diluent electrolyte than that in concentrated electrolyte. All these results indicated that the LiF-rich cathode-electrolyte interphase (CEI) formed on the surface of cathode (Fig. 8c), which is uniform and robust enough to protect the cathode materials from erosion. Xu and Zhang’s group developed a 4 M LiTFSI-LiDFOB-DME dual-salt electrolyte that induces the formation of stable EEI on both a high-voltage LiNi_1/3_Mn_1/3_Co_1/3_O_2_ cathode and the Li metal anode [34]. Transmission electron microscope (TEM) characterization exhibited the thinnest thicknesses of the CEI layers can be clearly observed on the cycled LiNi_1/3_Mn_1/3_Co_1/3_O_2_ (NMC) cathodes with concentrated dual-salt electrolyte. As a result, the prepared NMC||Li cell realized a capacity of ~ 80% and minimal increase of cell overpotential over 500 cycles with a cut-off voltage of 4.3 V (Fig. 8d). In addition, the apparent crystal boundary between CEI and NMC cathode was observed by atomic-resolution scanning transmission electron microscopy (STEM) and high-resolution transmission electron microscopy (HRTEM) in their following study. With an interlayer distance of 0.20 nm (Fig. 8e) [37], the crystalline phase was likely to be the LiF (*d*_200_ = 0.201 nm), which enable to form a highly inorganic CEI and achieves remarkable battery cycling stability with a capacity retention of ∼92% after 500 cycles (Fig. 8f).

HCEs can also significantly inhibit various adverse reaction including dendritic growth, Al corrosion, HRE, transition-metal dissolution, and polysulfides dissolution. Metal electrode is regarded as the ‘‘holy grail’’ material to realize the high-energy-density batteries due to its high specific capacity and low reduction potential. However, dendritic metal formation and uncontrolled interfacial reactions are major hurdles to the commercial application of metal batteries. Recent years on designing of the solvation structure of electrolyte was recognized to be an effectively way to alleviate the dendrite growth of the metal electrode. From previous reports, an unstable SEI on the metal anode is primarily responsible for dendrite growth and accompanied by low CE. In case of lithium metal, Jeong et al. first reported the phenomenon of suppressing dendritic lithium formation occurred in a 3.27 M LiTFS/PC electrolyte in 2008 [38]. TEM image of the cycled lithium electrodes was compared to reveal that the SEI on electrodeposited lithium is thinner and the dendritic lithium grew straighter in high-concentration solution than in low-concentration solution. And the Raman spectroscopy show that most of the PC molecules had solvated the Li^+^, which may contribute much to form robust SEI and suppress dendritic lithium formation. Suo et al. further demonstrated the suppression of lithium dendrite growth and shape change on the metallic lithium anode in a Li–S battery. They used a 7 M LiTFSI-DME electrolyte to significantly suppress the formation of dendritic lithium and enhance the cyclic performance of Li–S batteries. Moreover, the lithium polysulfide dissolution was also inhibited. Homogeneous deposition and dissolution of mental ions are believed to be a critical role to the stable operation of metal batteries, and the homogeneous coverage of the SEI film on anode is the prerequisite.

#### High Safety with Reduced Flammability

Meanwhile, the high volatility and flammability of organic solvents can be greatly overcome due to the much lower content of organic solvents in the concentrated solutions, which is favorable to future applications [18]. Yamada’s group paid much attention on this property of HCEs. They found that the super-concentrated 1:1.1 (molar ratio) LiFSA/DMC solution showed superior thermal stability and flame retardant ability when compared with the dilute electrolyte (1 M LiPF_6_-EC/DMC) [39]. They also demonstrated a concentrated solution using a salt and a flame-retardant solvent (trimethyl phosphate), without any additives or soft binders. The unusual passivation and fire-extinguishing properties of the 5.3 M LiFSA/TMP and 3.3 M NaFSA/TMP allow stable charge/discharge cycling for over 1000 cycles (over one year) with negligible degradation for graphite and hard carbon anodes, respectively. The reduction of EC solvent is the major route for the formation of passivation films in the 1.0 M NaPF_6_/EC: DEC. The resultant interface showed typical two-layer organic–inorganic hybrid structure, which is prone to grow into large particles rather than a compact uniform film, which failed to passivate the hard carbon anode. In sharp contrast, the reduction of FSA^−^ anions is dominant in the concentrated electrolyte of 3.3 M NaFSA/TMP. The decomposed products lead to a uniform inorganic passivation film with negligible organics detected. The EC-derived alkyl carbonate suffers from thermally unstable and can decompose at ~ 80 °C, diminishing the battery stability and safety. Specifically, the FSA^−^ derived inorganic SEI circumvented the poor passivation of common non-flammable solvents. Moreover, the strong interactions between the cations and solvent molecules can reduce the inherent volatility of the solvent. The thermogravimetric curves of various electrolytes indicated that the 3.3 M NaFSA/TMP showed high stability up to 150 °C, which is ascribed to the high boiling point of TMP and dominant Na^+^-TMP solvation with very limited free solvent molecules. This HCEs did not support combustion at all but serve as an efficient extinguisher of a fire. With the flame retardant TMP as sole solvent, the results shed light on a new feature of battery safety of HCEs [12].

### Local Highly Concentrated Electrolytes

As a prerequisite for forming LHCEs, diluent is an essential role to reduce the overall salt concentration but retain the highly concentrated salt-solvent clusters as they are in the HCEs. The following basic requirements should be considered choosing a diluent in LHCEs: (1) offer little or no solubility to cation mean that almost not changing the local coordination environment of HCEs; (2) chemical inertness with all components in battery system; (3) low volatility and low viscosity; (4) wide working temperature and low cost; (5) must be readily miscible with the solvating solvent in HCEs and form a uniform solution to avoid phase separation; (6) maintain as a non-flammability and environment friendly as possible to reduce safety risks and toxicity of electrolyte.

The design of LHCEs involves introducing inert diluents into HCEs to reduce the overall salt concentration while maintaining the essential solvation structure, thereby enabling both performance optimization and practical applicability. The core concept of LHCEs is the creation of a “locally concentrated” microenvironment, in which highly concentrated salt–solvent clusters (similar to the CIPs and AGGs observed in HCEs) are dispersed within low-viscosity diluents. Commonly used diluents, such as 2,2,2-trifluoroethyl methyl carbonate (FEMC), fluorinated ethers including bis(2,2,2-trifluoroethyl) ether (BTFE), 1,1,2,2-tetrafluoroethyl-2,2,3,3-tetrafluoropropyl ether (TTE), bis(2,2,2-trifluoroethyl) carbonate (BTFEC), and 2H,3H-decafluoropentane (HFC), possess weak solvating capabilities. These diluents can significantly reduce viscosity while maintaining chemical inertness and non-flammability. This design inherits the advantages of HCEs, such as a wide ESW, anion-derived interfacial passivation, and suppressed parasitic reactions. At the same time, it overcomes their major drawbacks by offering lower cost and better processability.

**Fig. 9:**
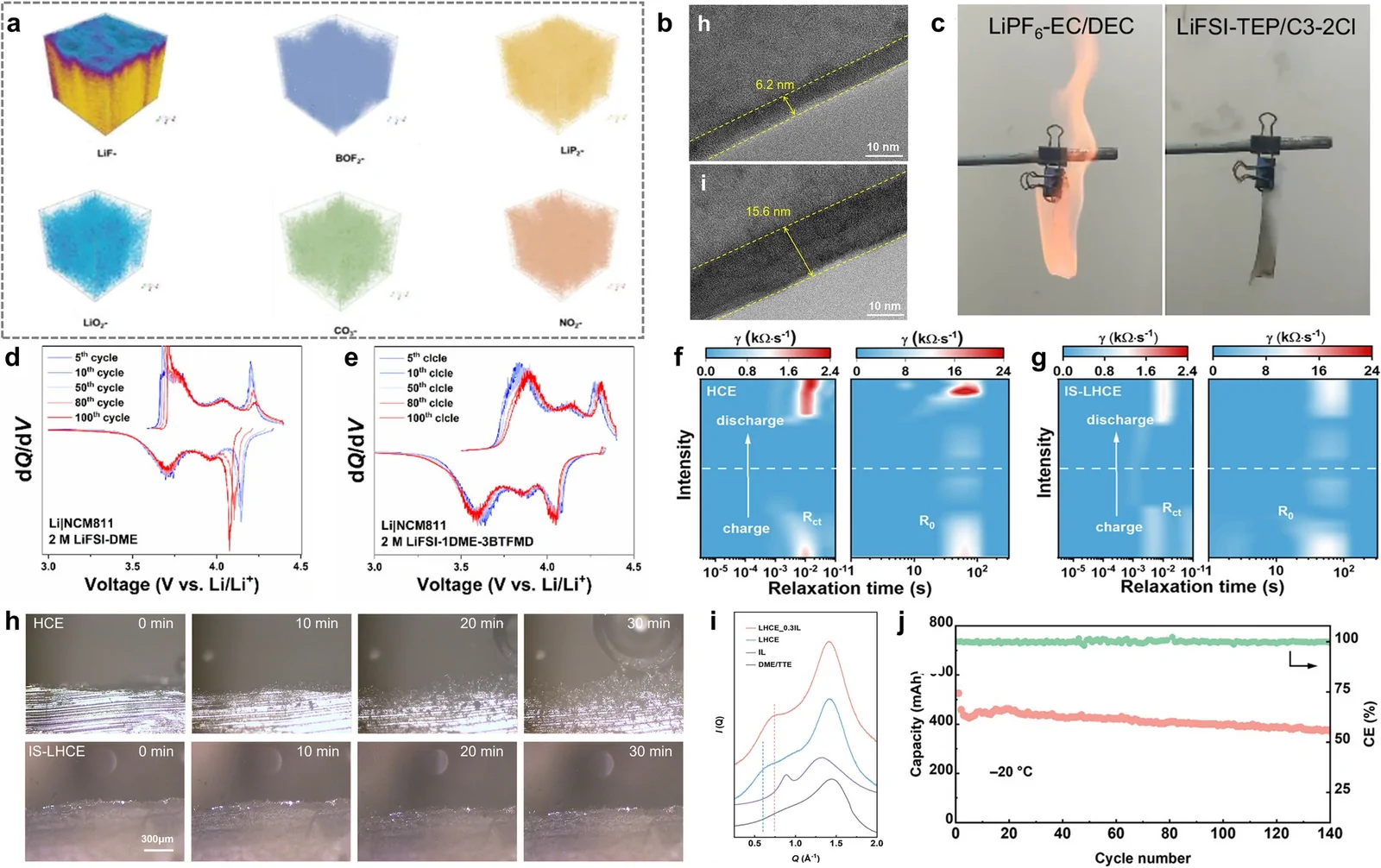
**a** TOF-SIMS of different substances. Reproduced with permission [40]. Copyright 2025, Wiley-VCH. **b** Cryo-TEM characterization of the morphology and thickness of the SEI layers formed on Li metal surface in different electrolytes. **c** Combustion tests of flammable cotton tapes soaked in different electrolytes. **b,** **c** Reproduced with permission [41]. Copyright 2025, Springer Nature. **d,** **e** dQ/dV profiles of Li||NCM811 full-cells at different cycles with 2 M LiFSI-DME and 2 M LiFSI-1DME-3BTFMD. **d,** **e** Reproduced with permission [42]. Copyright 2024, American Chemical Society. Cycling performance at − 60 °C and 0.2  C. DRT analysis in **f** HCEs and **g** IS-LHCEs. **h** In-situ optical microscopy observation of Na metal electrochemical deposition with HCEs and IS-LHCEs at 0.2 mA cm^−2^. **f–h** Reproduced with permission [43]. Copyright 2025, Wiley–VCH. **i** SAXS profiles for the solvents and electrolytes. **j** Pouch cell performance at − 20 °C and 0.1 C rate with the LHCEs-0.3IL electrolyte. **i, j** Reproduced with permission [44]. Copyright 2024, Wiley-VCH

Specifically, these diluents do not interfere with the LUMO/HOMO levels. As a result, the ESW of LHCEs can exceed 5.5 V (vs. Li^+^/Li), which enables preferential anion decomposition and the formation of robust SEI and CEI layer. Wang et al. reported a flame-retardant localized high-concentration gel polymer electrolyte (LHCE-GPE) [40], where a perfluorinated phosphazene diluent (PFPN) acted as a non-coordinating solvent. DFT, MD, and Raman analyses confirmed that PFPN did not participate in Li^+^ coordination, while the locally enriched anion environment strengthened Li^+^–anion coordination (TFSI^−^/DFOB^−^), forming an anion-rich solvation sheath. Because the diluent did not alter solvent redox levels, the anions (LiTFSI and LiDFOB) became the preferentially reduced species at the anode, yielding inorganic-rich interphases. TOF–SIMS mapping showed that Li-F and B-F were uniformly distributed throughout the SEI (Fig. 9a), whereas organic/carbonate components accumulated near the outer surface. Wu et al. employed DFT-MD simulations and data-driven modeling to reveal the heterogeneous EDL structure of a representative LHCEs (LiFSI salt, DME solvent [45], and TFEO diluent). They found that the EDL consisted of a Li⁺-rich salt–solvent cluster region and a Li^+^-depleted diluent-rich region. This structure preserved the micelle-like characteristics of LHCEs while introducing Li^+^ into the diluent region to screen charges, thereby increasing the reduction potential of TFEO and influencing SEI formation.

Moreover, LHCEs effectively suppress dendrite growth, aluminum corrosion, and transition-metal dissolution. These effects enhance battery cycling stability and safety. Wang et al. reported a flame-retardant LHCEs (LiFSI-TEP with a short-chain dichloroalkane diluent, C3-2Cl) that formed anion-dominated solvation sheaths and inorganic-rich interphases on both the anode and the cathode [41]. The weak coordination between C3-2Cl and Li^+^ improved ion transport and desolvation kinetics over a wide-temperature range. The LHCEs promoted anion-derived decomposition to form a thin (~ 6.2 nm) inorganic-rich SEI (Fig. 9b). This SEI reduced interfacial impedance and improved Li plating/stripping reversibility and kinetics. Meanwhile, LiFSI-TEP/C3-2Cl exhibited strong flame-retardant behavior (Fig. 9c). Lee et al. demonstrated that a fluorinated cyclic-ether diluent (BTFMD) enabled LHCEs that stabilized both Li metal anodes and high-voltage NCM811 cathodes [42]. Figure 9d, e showed that the BTFMD-based LHCEs maintained sharp and reversible dQ/dV peaks. It prevented the peak broadening near ~ 4.2 V that appeared in 2 M LiFSI–DME. This behavior indicated suppression of the irreversible phase transition and reduced cathode structural degradation and transition-metal dissolution, consistent with XRD (I(003)/I(104)) and LiF-rich CEI analysis.

This strategy is also applicable to sodium-ion battery. Wang et al. employed DOL as an in-situ polymerizable diluent to form a PDOL quasi-solid matrix [43]. This matrix maintained anion-rich LHCE solvation, reduced free solvent, and drove interfacial chemistry toward an inorganic NaF/Na_2_S-rich SEI. As shown in Fig. 9f, g, in-situ DRT measurements revealed stable, low, and uniform interfacial impedance. These results indicated that the PDOL-confined diluent stabilized interfacial kinetics and prevented impedance accumulation during cycling. In-situ optical microscopy (Fig. 9h) directly showed that dendrite formation was effectively suppressed in the IS-LHCEs electrolyte. As an efficient electrolyte-regulation strategy, LHCEs not only addressed the bottlenecks of traditional electrolytes but also provided a promising pathway toward sustainable next-generation energy storage technologies. Guo et al. used the ionic-liquid diluent N-propyl-N-methylpyrrolidinium bis(fluorosulfonyl)imide (C3mpyrFSI) to induce anion–solvent exchange [44]. This process reduced cluster size and strengthened Na^+^ coordination (Fig. 9i). At − 20 °C, the Na deposition overpotential decreased from 1.2 to 0.2 V. The ionic conductivity increased by 1.5–2 times. The resulting electrolyte enabled SPAN||Na cells to achieve a Coulombic efficiency above 99.8% and high sulfur utilization. A 0.5 Ah pouch cell also demonstrated stable cycling, significantly outperforming conventional LHCEs at low temperature (Fig. 9j). LHCEs not only address the bottlenecks of traditional electrolytes but also provide an innovative pathway toward sustainable next-generation energy storage technologies.

### Weakly Solvating Electrolytes

Weaken the solvation ability of solvent at molecular level is an effective electrolyte design strategy attractive features including high metal anode Coulombic efficiency, inhibiting the dendritic growth/parasitic reactions, and fast desolvation kinetics at low temperature. In fact, although WSEs with a weakly interacts between ions and solvent, this does not imply that the concentration of WSEs is similar to that of conventional dilute electrolyte. Conventional electrolyte typically contains a large number of SSIPs structure, and the anions are almost outside the primary solvation sheath of the cations. In contrast, weakly solvating electrolyte was prone to display a characteristic CIPs or AGGs structure, in which the cation solvation sheath comprises both anions and solvent molecules. Unlike the solvent dominated solvation structure in conventional dilute electrolytes, the unique solvation structure of WSEs show an enhanced cation–anion interaction due to the weak solvent binding, which could lead to preferential reduction of anions to improved stability of EEI [46–49].

**Fig. 10:**
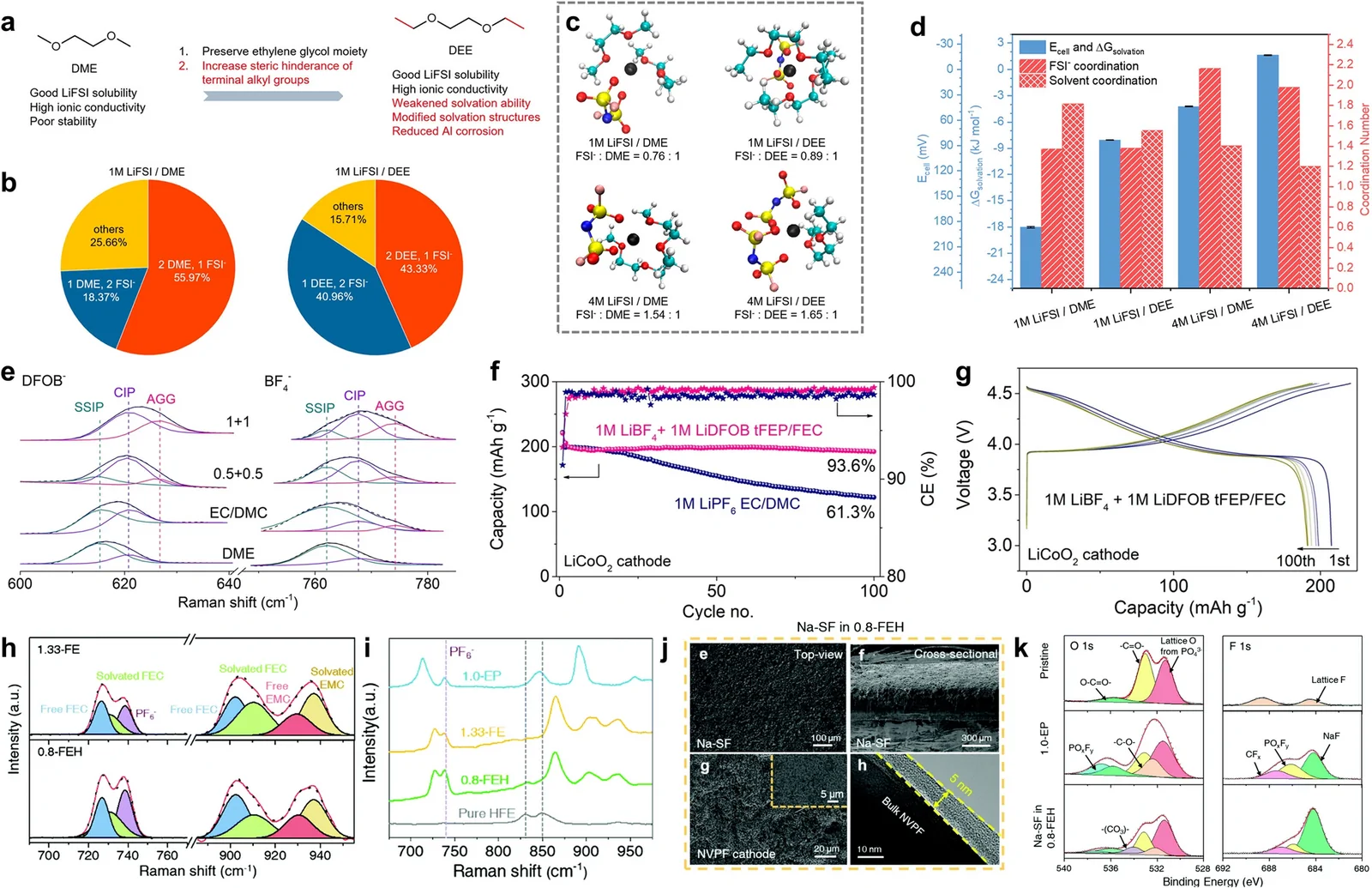
**a** Hypothesized molecular design utilizes steric hindrance effect from the end substituents to tune the solvation properties of solvent molecules. **b** Distributions of possible inner solvation shell compositions of 1 M LiFSI/DME and DEE from MD simulation. **c** Left *Y-*axes: open circuit voltages (*E*_cell_) and corresponding solvation energies (Δ*G*_solvation_) of the electrolytes (blue); right *Y*-axis: number of FSI^–^ (red slashes) and solvents (red crisscross) in the inner solvation shell. *E*_cell_ and Δ*G*_solvation_ values are in reference to 1 M LiFSI in DEC. **d** Structures of the most probably inner solvation shells and average FSI^−^ to solvent ratios of the four electrolytes from MD simulation. **a–d** Reproduced with permission [46]. Copyright 2021, American Chemical Society. **e** Raman spectra for DFOB^−^ and BF_4−_ anions with LiDFOB and LiBF4 dissolved in various solvents. **f** Cycling performance and CE of LiCoO_2_ cathode at 0.5 C. **g** Corresponding voltage profiles of LiCoO_2_ cathode in 1 M LiBF_4_ + 1 M LiDFOB tFEP/FEC electrolyte. **e–g** Reproduced with permission [50]. Copyright 2023, Springer Nature. **h** Raman spectra of the corresponding electrolyte from 675 to 975 cm^−1^. **i** Fitting results of the Raman spectra of the 1.33-FE and 0.8-FEH electrolytes. **j** Top-view and cross-sectional SEM images of the Na-SF electrode cycled in the 0.8-FEH electrolyte. **k** Deconvoluted C 1*s*, O 1*s* and F 1*s* XPS spectra of the pristine NVPOF cathode and the NVPOF cathode after 100 cycles in the 1.0-EP electrolyte and the 0.8-FEH electrolyte. **h–k** Reproduced with permission [51]. Copyright 2021, Royal Society of Chemistry

Increasing steric effect of molecular is an effective approach to weakend solvation ability of solvent. For example, by replacing the terminal methoxy groups of DME to ethyl groups, the resulting 1,2-diethoxyethane (DEE) still peserved desirable chelation with Li^+^ and consequently sufficient solubility of Li salt for high ionic conductivity. Based on this design principle, Bao’s group adopted steric hindrance to tune the solvation ability of ether solvents. influence of steric hindrance effect produced by DME and DEE molecules with different solvation abilities on electrochemical performance of Li metal batteries [46]. Compared with DME molecules, DEE molecules with two terminal methoxy groups have more sterically hindered functional groups (Fig. 10a, b). Although the calculated result of the binding energy between one solvent molecule with one Li^+^ indicates that the binding energy is slightly higher for DEE than DME (Fig. 10c). At both high and low concentration, there are fewer DEE than DME molecules in the Li^+^ solvation shell was demonstrated by ^7^Li NMR, Raman spectra and solvation energy measurements (Fig. 10d). Thus, the apparent weaker solvation ability of DEE compared to DME in solution may not come from the difference in Lewis basicity of the oxygen atoms in DEE to DME, but rather originates from the increased steric hindrance of ethoxy groups compared to methoxy groups. As a result, the DEE-based electrolyte exhibits higher Li Coulombic efficiency and no Al corrosion at high voltage. In another study, Mao et al. constructed a weakly solvating electrolyte by combining a fluorinated linear carboxylate ester (ethyl 3,3,3-trifluoropropanoate, tFEP) with a weakly coordinating co-solvent (FEC) and weakly dissociated lithium salts (LiBF_4_ and LiDFOB) [50]. This molecular design enabled anion incorporation into the primary solvation sheath even at low salt concentrations, thereby promoting the formation of a high fraction of CIPs/AGGs and facilitating the generation of a LiF-rich interphase (Fig. 10e). The optimized electrolyte exhibited excellent compatibility with high-voltage 4.6 V LiCoO_2_, retaining ~ 93.6% of the initial capacity after 100 cycles (Fig. 10f, g), in stark contrast to only ~ 61.3% retention in the conventional 1 M LiPF_6_ EC/DMC electrolyte. This strategy highlighting its markedly enhanced high-voltage cycling stability.

**Fig. 11:**
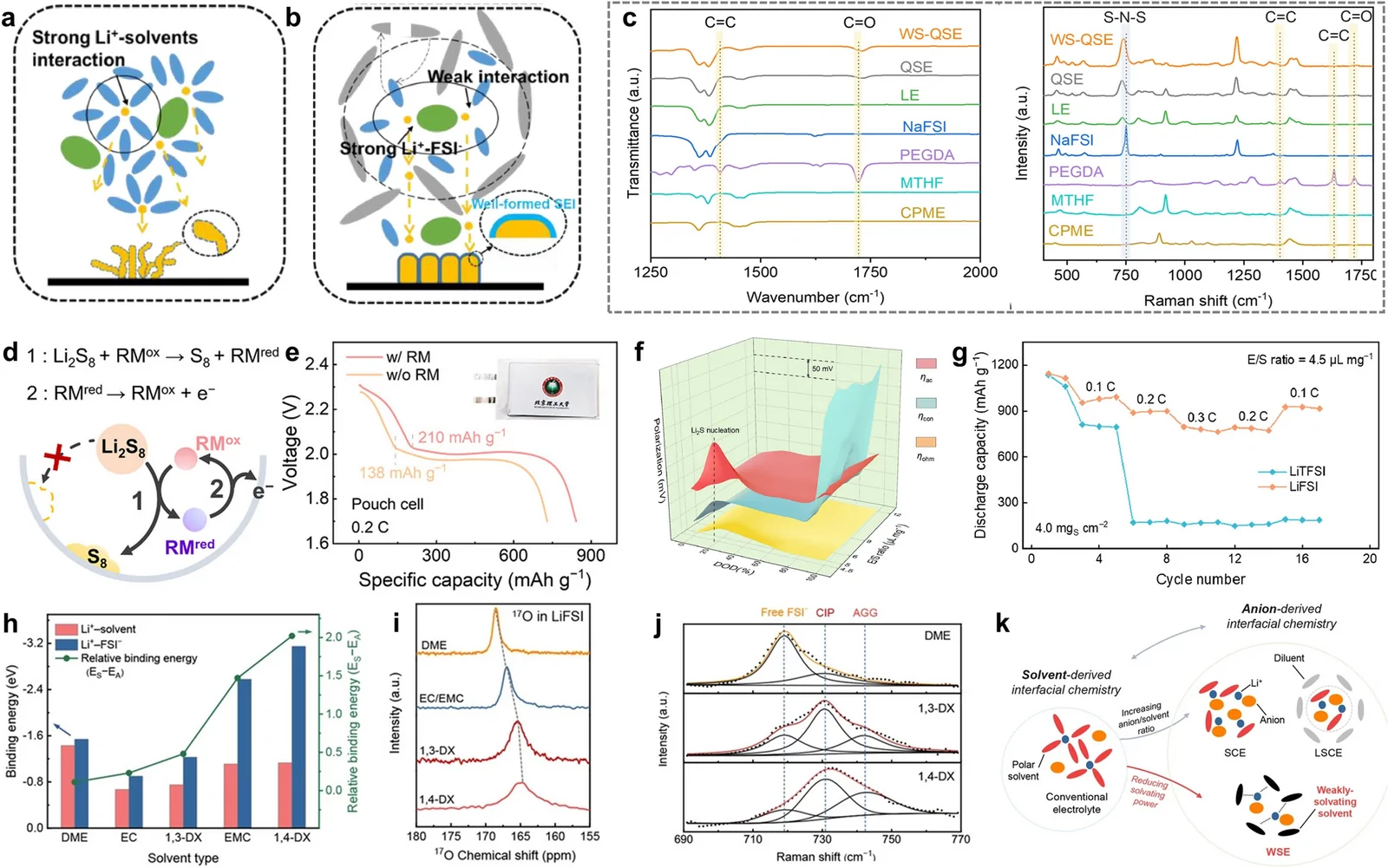
**a,** **b** Illustration of Li plating/stripping moderated by solvent structure and SEI. **a,** **b** Reproduced with permission [52]. Copyright 2022, Elsevier. **c** ATR-FTIR and Raman spectroscopy characterization of WS-QSE, QSE, LE, NaFSI, PEGDA, MTHF, and CPME. Reproduced with permission [53]. Copyright 2025, Royal Society of Chemistry. **d** Schematic illustration of the Li_2_S_8_ oxidation pathway with or without the charging RM in WSEs-based Li‒S batteries. **e** Charge‒discharge profiles of Li‒S pouch cells with or without the charging RM at 0.2 C. **d**, **e** Reproduced with permission [54]. Copyright 2025, Wiley-VCH. **f** Variation of *η*_ac_, *η*_con_, and *η*_ohm_ along the depth of discharge (DOD) and different E/S ratios. **g** Rate performances of weakly-solvating Li‒S cells at the E/S ratio = 4.5 μL mg^−1^. **f,** **g** Reproduced with permission [55]. Copyright 2025, Elsevier. **h** Binding energies between Li^+^ and solvents/anions obtained by first-principles calculations. **i** Natural-abundance ^17^O NMR spectra of 1.0 M LiFSI dissolved in various solvents. Signals were collected at 60 °C. **j** Raman spectra of 1.0 M LiFSI dissolved in various solvents. **k** Solvation structures in conventional electrolyte, superconcentrated electrolyte (SCE), LSCEs, and WSEs. **h–k** Reproduced with permission [49]. Copyright 2021, Wiley-VCH

Apart from that, the addtion of non-solvating molecular can also weaken solvent participation in the cation solvation structure. Zheng et al. tailored a novel Na-baesed electrolyte for Na_3_V_2_(PO_4_)_2_O_2_F||Na cell at low temperature, consisting of 0.8 M NaPF_6_ in FEC/EMC/HFE (3:3:4 by vol.) (denoted as 0.8-FEH) [51]. In this design, the linear structured EMC and cyclic structured FEC are used to dissolve Na salt, while the non-solvating, highly-fluorinated HFE is used to further weaken the electrolyte affinity with Na^+^ ions, which can be confirmed by the blueshift of solvated FEC and EMC in Raman spectra (Fig. 10h, i). As a result, the cycled Na_3_V_2_(PO_4_)_2_O_2_F cathode in 0.8-FEH maintained a highly-uniform, much thinner and compact surface can be observed in SEM and HRTEM images (Fig. 10j). XPS spectra was carried out to prove the chemical compositions are NaF-enriched and highly-inorganic (Fig. 10k). Benefit from the fast ion de-solvation process of WSEs and stable EEI, the assembled Na_3_V_2_(PO_4_)_2_O_2_F||Na cell shows a high-capacity retention of 89.2 and 92.1 mAh g^−1^, respectively, under ultra-high rate of 30 C and low temperature of − 30 °C. Based on the similar mechanisms, Zhu et al. reported a WSEs of 1 M LiFSI-DOL/TTE (1:2) which can enable SPAN||Li cell to deliver highly reversible Li plating/stripping behavior with an average CE of 99.77%. Compared to the tranditional electrolyte with strong Li^+^-solvent interaction, the addition of TTE weaken Li^+^-solvent interaction to reshape the Li^+^ coordination, thereby blocking the decomposition of DOL and mitigating preferential lithium nucleation (Fig. 11a, b) [52]. As a result, during the process of Li plating/stripping, the plated Li was mainly converted into uniform and compact lumps in WSEs and fewer dead Li was observed in Cu surface. Law et al. introduced a weakly solvating quasi-solid electrolyte (WS-QSE) for sodium metal batteries (NMBs) [53]. The WS-QSE was fabricated via in-situ polymerization of polyethylene glycol diacrylate (PEGDA) with 2 M sodium bis(fluorosulfonyl)imide (NaFSI) in a mixed solvent of 2-methyltetrahydrofuran (MTHF) and cyclopentanone methyl ether (CPME). As shown in Fig. 11c, the characteristic shift of the C = O stretching band indicated that the C-O bonds on the PEGDA backbone weakened Na^+^-solvent coordination. This effect promoted anion-dominated solvation structures and enabled the formation of a robust anion-derived SEI.

Beyond this, CEI serves as the core interfacial layer between the cathode and electrolyte. It directly governs charge-transfer efficiency, active-material utilization, and interfacial stability, and thereby the rate capability, cycle life, and energy density of the battery. WSEs not only overcome the intrinsic shortcomings of traditional electrolytes but also offer unique advantages for precisely tuning the CEI. In Li–S batteries, the main challenge lies in LiPS shuttle-induced anode corrosion, while strongly solvating electrolytes (SSEs) facilitate rapid sulfur redox but sacrifice cycling stability. Although WSEs suppress LiPS dissolution and shuttle, they tend to cause the accumulation of unreacted low-order LiPSs or insulating Li_2_S within the CEI, resulting in sluggish cathode kinetics. To resolve this contradiction, Jin et al. designed a “WSEs + redox mediator (RM)” electrolyte system [54]. Based on a DOL/DME-LiTFSI SSE, HME was used to construct a WSEs, and benzo[1,2-b:6,5-b]dithiophene-4,5-dione (BDTD) was introduced as the RM. The weak solvation of WSEs effectively suppressed LiPS shuttle and protects the Li anode. Meanwhile, BDTD established a chemical oxidation pathway converting LiPSs into S₈ followed by electrochemical regeneration of the RM (Fig. 11d), thereby reducing the accumulation of inert species in the CEI. As shown in Fig. 11e, a 3 Ah pouch cell operated at 0.2 C delivered a high upper-plateau capacity of 210 mAh g^−1^. Under lean-electrolyte conditions, where CEI kinetics became even more restrictive, their group subsequently developed a DIPS-based WSEs using diisopropyl sulfide (DIPS) [55], a low-donor-number co-solvent, and replaced LiTFSI with lithium bis(fluorosulfonyl)imide (LiFSI). The DIPS-based WSEs formed an “inner-shell dissolution–outer-shell protection” configuration that confines LiPSs and suppresses shuttle. As shown in Fig. 11f, GITT-EIS decoupling clearly revealed a sharp increase in activation polarization (η_ac_) during Li₂S nucleation as the E/S ratio decreases. This result identified CEI charge transfer as the dominant kinetic barrier in lean electrolytes. To address this issue, LiFSI modulated the CEI solvation structure through anion coordination with LiPSs. As validated by the rate performance in Fig. 11g, at an ultra-low E/S ratio of 4.5 μL mg^−1^, cells using LiFSI–WSEs maintained a discharge capacity of 798 mAh g⁻^1^ at 0.3 C, whereas LiTFSI-based electrolytes remained functional only at 0.1 C, demonstrating these results demonstrated that CEI optimization effectively lowered charge-transfer resistance and overcame the kinetic limitations imposed by lean-electrolyte conditions. This highlighted the substantial potential of WSEs for performance enhancement through precise CEI regulation.

In fact, quantifying and predicting the influence of ions and solvents on the solvation sheath is instrumental to design more applicable electrolyte. By calculating the difference of the binding energy between Li^+^-solvent (E_S_) and Li^+^-anion (E_A_), Yao et al. proposed a quantitative indicator to predict the extent of anion invasion into primary solvation sheath of Li^+^ [49]. As shown in Fig. 11h, the trend of E_S_-E_A_ follow the oder of DME < EC < 1,3-dioxane (1,3-DX) < 1,4-dioxane (1,4-DX), which is complete concordance with results of Raman and ^17^O NMR spectra (Fig. 11i, j). The DME-based electrolyte with the highest relative binding energy show the greatest free anions of FSI^−^ in Raman spectra, indicating that the majority of anions are excluded from the primary solvation sheath of Li^+^ due to the strong solvating power of DME. In contrast, 1,4-DX-based electrolyte with lowest relative binding energy exhibit fewest free anions of FSI^−^ while the highest CIPs and AGGs, accordingly, smaller shielding effect on the electronic environment of ^17^O nuclei of FSI^−^ was observed in ^17^O NMR spectra. It is worth noting that the trend of E_S_-E_A_ of the used solvent is quite different from the decreasing order of dielectric constant (*ε*_EC_ = 89.9, *ε*_EMC_ = 2.9, *ε*_DME_ = 7.2, *ε*_1,3-DX_ = 13.0, *ε*_1,4-DX_ = 2.2), which is also an important indicator to compare the dissolvability of solvent. This is because a high ε is not enough to guarantee a highly-solvating solvent. For example, nitromethane has a large dipole moment and hence a high *ε* of 38.6, yet it is a poorly-solvating solvent. The solvent molecule should have an ability to interact with ions, typically cations, sufficiently strongly in energy terms to overcome the lattice energy of salt. As a result, the unique solvation structure of WSEs leads to preferential reduction of anions to from derived and inorganic-rich SEI, which enable high-rate and long-term cycling of LIBs (Fig. 11k).

### H-Bond Regulation

#### Aqueous Electrolytes

**Fig. 12:**
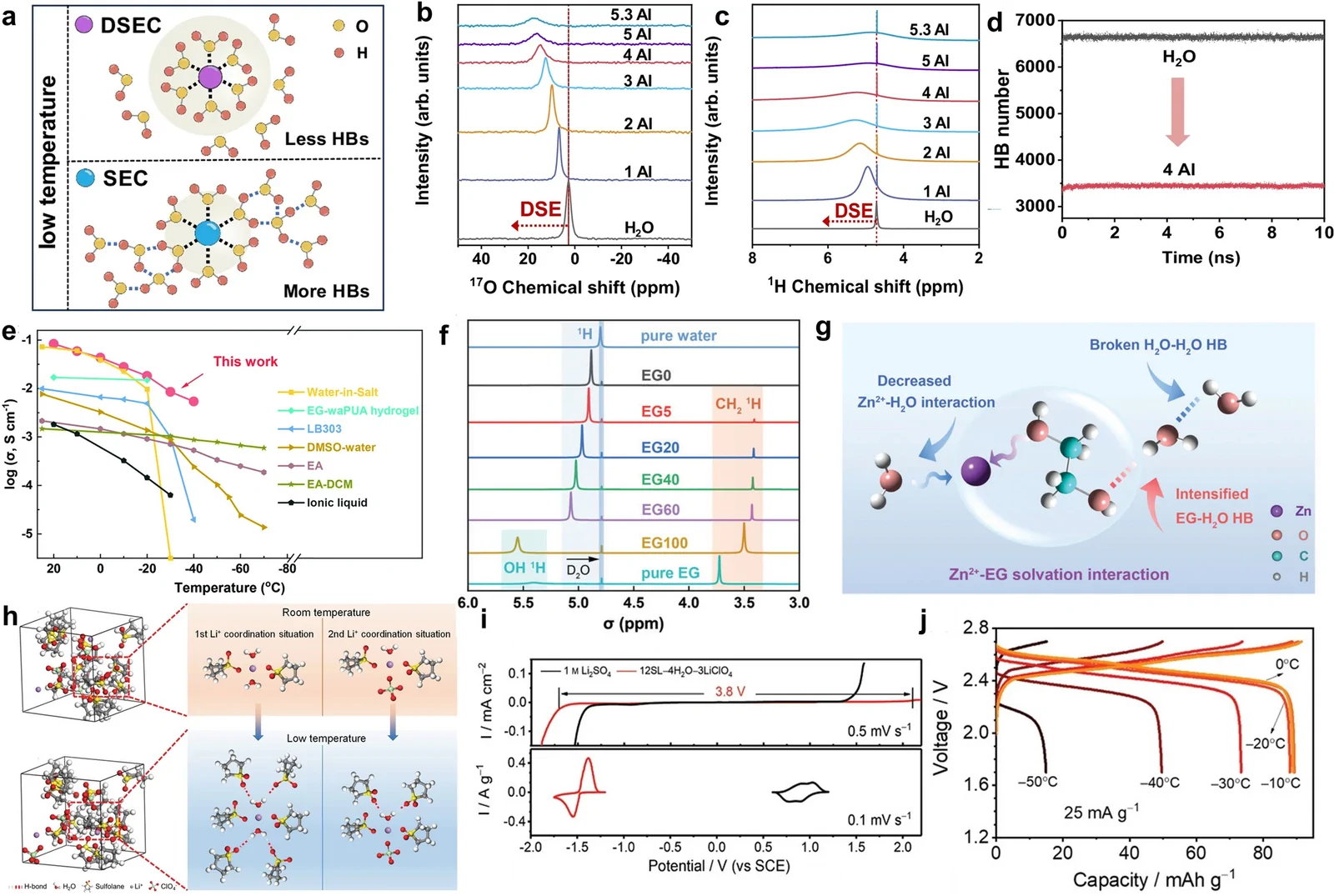
**a** Design concept for different types of cations to interact with water and decrease the number of HBs among water molecules. **b** ^17^O NMR and **c** ^1^H NMR spectra of different AlCl_3_ concentration electrolytes. **d** Calculated HB number between water molecules. **a–d** Reproduced with permission [56]. Copyright 2025, Springer Nature. **e** Comparison of the hybrid electrolytes with various other low-temperature electrolytes. **f** ^1^H NMR spectrum of different electrolytes at 25 °C. **g** Schematic illustration of a possible mechanism of how the Zn^2+^-EG solvation interaction impacts the chemistry of the hybrid electrolyte. **e–g** Reproduced with permission [57]. Copyright 2020, Royal Society of Chemistry. **h** MD stimulations of 12SL-4H_2_O-3LiClO_4_ electrolyte. **i** LSV curve of 12SL-4H_2_O-3LiClO_4_ electrolyte and 1 M Li_2_SO_4_ solution, CV curves of LMO and LTO operated in 12SL-4H_2_O-3LiClO_4_ electrolyte. **j** Electrochemical performance of LMO/LTO full battery using 12SL-4H_2_O-3LiClO_4_ electrolyte at − 20 °C. **h–j** Reproduced with permission [58]. Copyright 2022, Wiley-VCH

H-bond is a common electrostatic force which plays an essential role in the physicochemical of liquid water. However, the abundant H-bond interactions between water molecules cause a high freezing point that severely hinder its further application in cold regions. The introduction of H-bond acceptor could regulate the H-bond quantity and reduce the highly H-bound water molecules which can validly suppress the freeze of water in kinetic pathway. Recently, Feng et al. proposed a bidirectional H-bond regulation strategy enabled by cations to achieve ultra-low-temperature operation. High-charge-density Al^3+^ acted as a “de-shielding cation” (DSEC) [56]. It removed electron shielding around both O and H atoms in water molecules. thereby significantly weakening the strength and quantity of intermolecular H-bond (Fig. 12a). As shown in Fig. 12b, c, with increasing AlCl_3_ concentration (1–5.3 m), the ^17^O NMR signals exhibited continuous downfield shifts, while the ^1^H chemical shifts showed a pronounced downfield movement in the 1–4 M range, indicating reduced electron density and a simultaneous loss of H-bond donor and acceptor capability for both O and H atoms. MD simulations (Fig. 12d) further revealed that the number of H-bond between water molecules in the Al^3+^-based system was substantially lower than in pure water and remained stable over time, confirming the persistent disruption of the H-bond network. Remarkably, only 2.8 M Al^3+^ was required to depress the freezing point to − 117 °C, which enabled Zn||PANI pouch cells to retain 100% capacity after 500 cycles at − 70 °C. The similar mechanism was also reporeted in the ethylene glycol (EG)-water system by Chang et al. [57]. The mixed solvent show a high zinc-ion conductivity of 6.9 mS cm^−1^ at − 40 °C high reversibility of Zn plating/stripping (Fig. 12e). With the increase of the EG content for EG-water hybrid solvents, a slight chemical shift of ^1^H from H_2_O corresponding to the H-bond of EG-water, which illustrate the H-bond of EG-water is improved and the H-bond of water-water is greatly broken (Fig. 12f, g), thus providing the hybrid electrolyte with a lowered freezing point and high ionic conductivity even at -40 °C. Liu et al. selected sulfolane (SL) as co-solvent to prepare a high voltage and ultralow-temperature aqueous LIB [58]. The MD simulations results revealed that here is strong interaction between SL and water molecules and few water molecules uncoordinated with Li^+^ ions within LiClO_4_-SL/H_2_O electrolyte (Fig. 12h) [58]. That means the uncoordinated water molecules are not dissociative, which are bound with SL molecules. As a result, this LiClO_4_-SL/H_2_O effectively show a wide ESW of 3.8 V and ultra-low glass-transition temperature of − 110 °C (Fig. 12i, j).

**Fig. 13:**
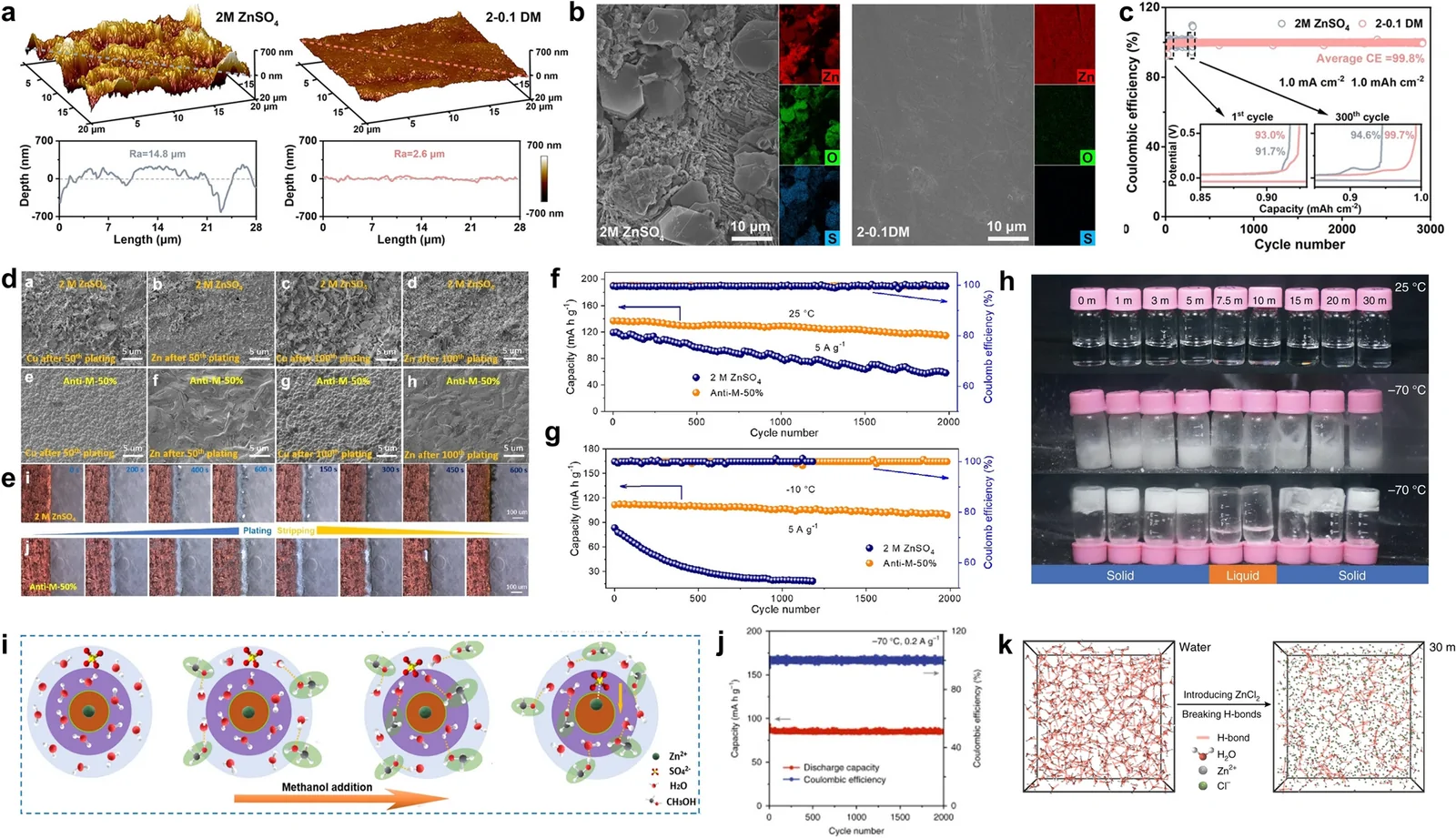
**a** AFM images with corresponding roughness. **b** SEM images with EDS mapping of Zn electrodes after 100 cycles. **c** CE of the long-time performance with a cutoff voltage of 0.5 V. (**a**–**c**) Reproduced with permission [59]. Copyright 2025, Wiley–VCH. **d,** **e** SEM and in-situ optical microscopy studies on Zn plating behavior. **f**, **g** Cycling stability of Zn/PANI coin cells at 5 A g^−1^ with different electrolytes. **h** Optical photographs of different electrolyte at 25 and − 70°C **i** Snapshot of the MD simulation of water and 30 m ZnCl_2_ electrolyte. The red line represents the H-bonds. **j** Cycling performance at − 70°C and 0.2  A g^−1^. **f**–**h**, **j** Reproduced with permission [61]. Copyright 2020, Springer Nature. **k** Schematic of changes in the Zn^2+^ solvent sheath, together with methanol addition. **d,** **e,** **i,** **k** Reproduced with permission [60]. Copyright 2021, Wiley-VCH

Besides, the strategies of H-bond regulation was also investigated as a candidate for prevention of the side reactions and dendrite growth because of the strong H-bond interaction between water and organic solvent can inhibit these bad effects. Recently, Wang et al. proposed a targeted localized H-bond docking mechanism by introducing polyhydroxy hexitols as electrolyte additives [59]. Because hexitols acted as both H-bond donors (H in -OH) and acceptors (O in –OH), they interacted dynamically with surrounding water molecules and disrupted the intrinsic tetrahedral H-bond network. This disruption lowered water activity and suppressed proton transfer, thereby fundamentally mitigating the hydrogen evolution reaction (HER) and the formation of zinc hydroxysulfate (ZHS) in AZIBs. As shown in Fig. 13a, b, AFM and SEM analyses revealed that the Zn anode cycled in the hexitol-containing electrolyte exhibited a smooth and compact surface without flake-like ZHS by-products, in sharp contrast to the rough, heavily passivated surface observed in the baseline electrolyte. Furthermore, Fig. 13c demonstrated excellent Zn reversibility, with a Coulombic efficiency of 99.8% and a symmetric cell lifespan exceeding 5000 h. Hao et al. [60] demonstrated that methanol as a co-solvent to regulate aqueous ZnSO_4_ electrolyte for boost Zn chemistry reversibility. With the H-bond formation between methanol molecules and water molecules, methanol gradually lead a dominance of outer and inner sheath of Zn^2+^ solvation (Fig. 13i). And methanol molecules are more likely to adsorb on Zn metal surfaces to impact water-induced H_2_ evolution and Zn nucleation formation during Zn plating/stripping. The SEM images and in-situ optical microscope images of Cu electrode during Zn plating/stripping show a good reversibility of Zn plating/stripping with uniform spherical deposition and non-dendrite growth in ZnSO_4_-H_2_O-methanol (contains 50 v/v% methanol) electrolyte (Fig. 13d, e). As a result, the assmbled Zn||polyaniline (PANI) cells show a excellent cycliability in ZnSO_4_-H_2_O-methanol electrolyte at both room and low temperature, with a capacity retention of 85.5% (25 °C) and 89.3% (− 10 °C) after 2000 cycles (Fig. 13f, g).

**Fig. 14:**
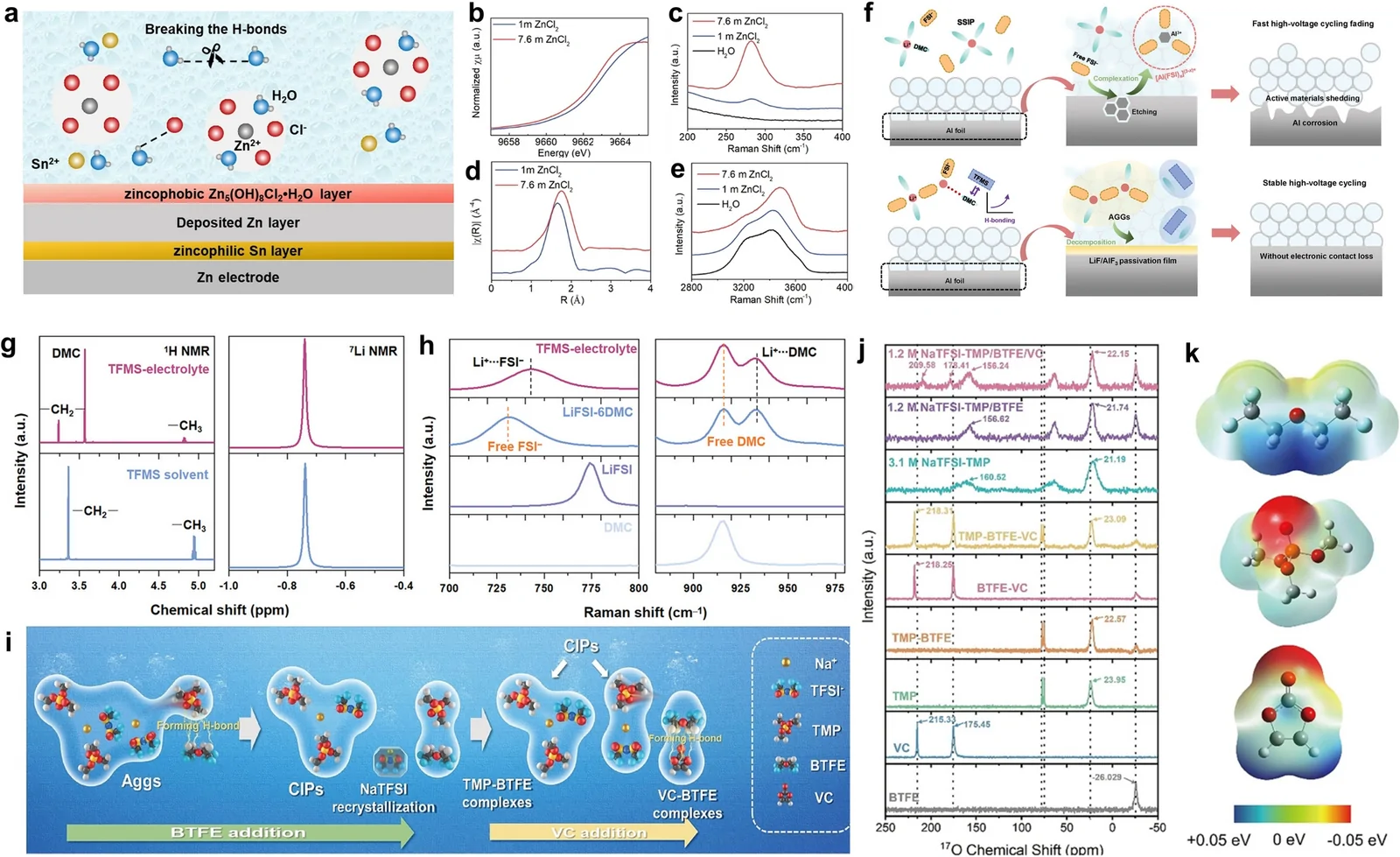
**a** Scheme of electrolyte and electrolyte–electrode-interphase structure. **b–e** Physical properties and structure of ZnCl_2_ aqueous electrolytes. **a–e** Reproduced with permission [62]. Copyright 2021, Wiley-VC. **f** Schematic illustrations of the Al-CC corrosion in LiFSI-6DMC electrolytes without and with TFMS addition. **g** ^1^H-NMR and ^7^Li-NMR spectra of TFMS-electrolyte and TFMS solvent. **h** Raman spectra of the TFMS-electrolyte, LiFSI-6DMC, LiFSI, and DMC. **f–h** Reproduced with permission [65]. Copyright 2024, Wiley-VCH. **i** Illustration of changes in the electrolyte structure with the addition of BTFE and VC. **j** Natural-abundance ^17^O-NMR spectra of different solvents and electrolytes. **k** ESP comparison of BTFE, TMP, and VC. **i–k** Reproduced with permission [67]. Copyright 2021, Wiley-VCH

Except the formation of new H-bond between water and added solvent, the breakge of original H-bond network by modulating the salt concentration was also an effective strategy to improve electrochemical performance for aquoues battery. In principle, more H-bonds in water will be destroyed with the increase of salt concentration. However, the excessive dissolved salt at room temperature will precipitate at low temperature, which limits the liquid temperature range [61, 62]. Chen’s group investigated a series of solid–liquid transition states of ZnCl_2_-water electrolyte with different concentrations (from 0 to 30 mol kg^−1^) at room temperature and − 70 °C [61]. As shown in Fig. 13h, the ZnCl_2_-water electrolyte with a moderate ZnCl_2_ concentration (7.5–10 mol kg^−1^) show the best flowability at − 70 °C. The mechanism was demonstrated by the structural and spectroscopic studies combined with theoretical calculation. With the addition of ZnCl_2_, the peaks of O–H stretching vibration of water molecules shift to high frequency, indicating that the proportion of H-bond in ZnCl_2_-water elelctrolyte decreased significantly. The results of MD simulation reveled that liquid phase show the massive reduction of H-bonds by inducing ZnCl_2_ (Fig. 13k). As a result, the cycling performance of the assembled Zn||PANI pouch cell could achieve 2000 cycles with the capacity retention of ~ 100% at 0.2 A g^−1^ (Fig. 13j). Based on a similar mechanism, Cao et al. reported a 7.6 M ZnCl_2_ + 0.05 M SnCl_2_ aqueous electrolyte, which could overcome the obstacle of Zn dendritic growth and poor low temperature (Fig. 14a) [62]. The disruption of the water network and electrolyte structure was further confirmed by X-ray absorption spectroscopy (XAS), X-ray absorption near edge structure (XANES) spectra, NMR and Raman. The disappearance of free water and H-bond reduce the reactivity of H_2_O and improve the cycling performance, leading to increased zinc plating/stripping efficiency (Fig. 14b–e).

#### Non-Aqueous Electrolytes

Except the H-bond regulation for aqueous electrolyte, this strategy was also used in non-aqueous electrolyte. The addition of H-bond can alter the LUMO energy level of anions and reduce the amount of free solvent molecules in electrolyte, which could promote the formation of robust SEI and enhance the stability of electrolytes. Especially for strongly electron-donating molecules contains partially positively charged atoms (such as H) or partially negatively charged atom (such as O and F), which are easy to form strong or moderate interaction between other polar molecules. For example, EC was found to be able to bond with F atom on the anion, such as PF_6_^−^ and BF_4_^−^ [63, 64]. The weak anion-solvent interactions between EC and PF_6_^−^ could provide protection against the decomposition of EC on cathode surface and current collector. Cresce et al. concluded that F atom in these anions are prone to from intensely interaction with the weakly electropositive regions in solvent molecules by using electrospray-ionization mass spectrometry (ESI–MS), NMR and computational simulation [63]. The H-bond interaction weaken the effect of solvent on the electrochemical behavior on cathode surfaces compared to the effect of the competitive solvation of solvent to Li^+^ on the SEI formation on anode. Zhang et al. found that introducing the co-solvent 2,2,2-trifluoroethyl methanesulfonate (TFMS) enabled H-bond interactions with dimethyl carbonate (DMC) [65], thereby regulating the Li^+^ solvation structure in LiFSI-based electrolytes (Fig. 14f). As shown in the ^1^H NMR spectra in Fig. 14g, the two characteristic proton signals of TFMS exhibited an upfield shift after the addition of LiFSI–6DMC electrolyte, which arose from the shielding effect induced by the strong H-bond interactions between DMC and TFMS. In contrast, the ^7^Li NMR spectra showed negligible changes in the chemical shift of Li^+^ (Fig. 14h), indicating that H-bond regulation only altered the relative ratio of DMC and FSI^−^ within the solvation shell without disrupting the primary Li^+^ coordination structure, thus maintaining the stability of the Li^+^ solvation environment. Compared with the LiFSI–6DMC electrolyte, the characteristic band at 743.2 cm^−1^ was significantly intensified in the TFMS-containing electrolyte, suggesting a substantial increase in the fraction of anions participating in the Li^+^ solvation sheath. Based on this H-bond regulation strategy, 1.2 Ah graphite||NCM811 pouch cells demonstrated outstanding high-voltage performance, retaining 89.9% of their capacity after 200 cycles at 4.4 V with an average Coulombic efficiency above 99.9%, while exhibiting neither noticeable Al current-collector corrosion nor gas evolution. A similar phenomenon was also observed in a non-flammable electrolyte (1.2 M NaTFSI-TMP/BTFE/VC) by Yang et al. [66]. The addtion of non-solvating molecular BTFE reduced the viscosity and concentration of the electrolyte, however, the fromation of H-bond between BTFE and TMP leads to the destruction of Na^+^-TMP solvation structure. They choose a highly polar molecule VC as recovery solvent to banlance the damaged solvation structure, due to the fromation of H-bond between BTFE and VC can alleviate the effect of BTFE on TMP (Fig. 14i) [67]. As evidenced by spectrographic analysis and computational simulation, the O atom on TMP and VC can interact with H atom on BTFE to form BTFE-TMP and BTFE-VC complexes by H-bond (Fig. 14j, k).

### Eutectic Electrolytes

**Fig. 15:**
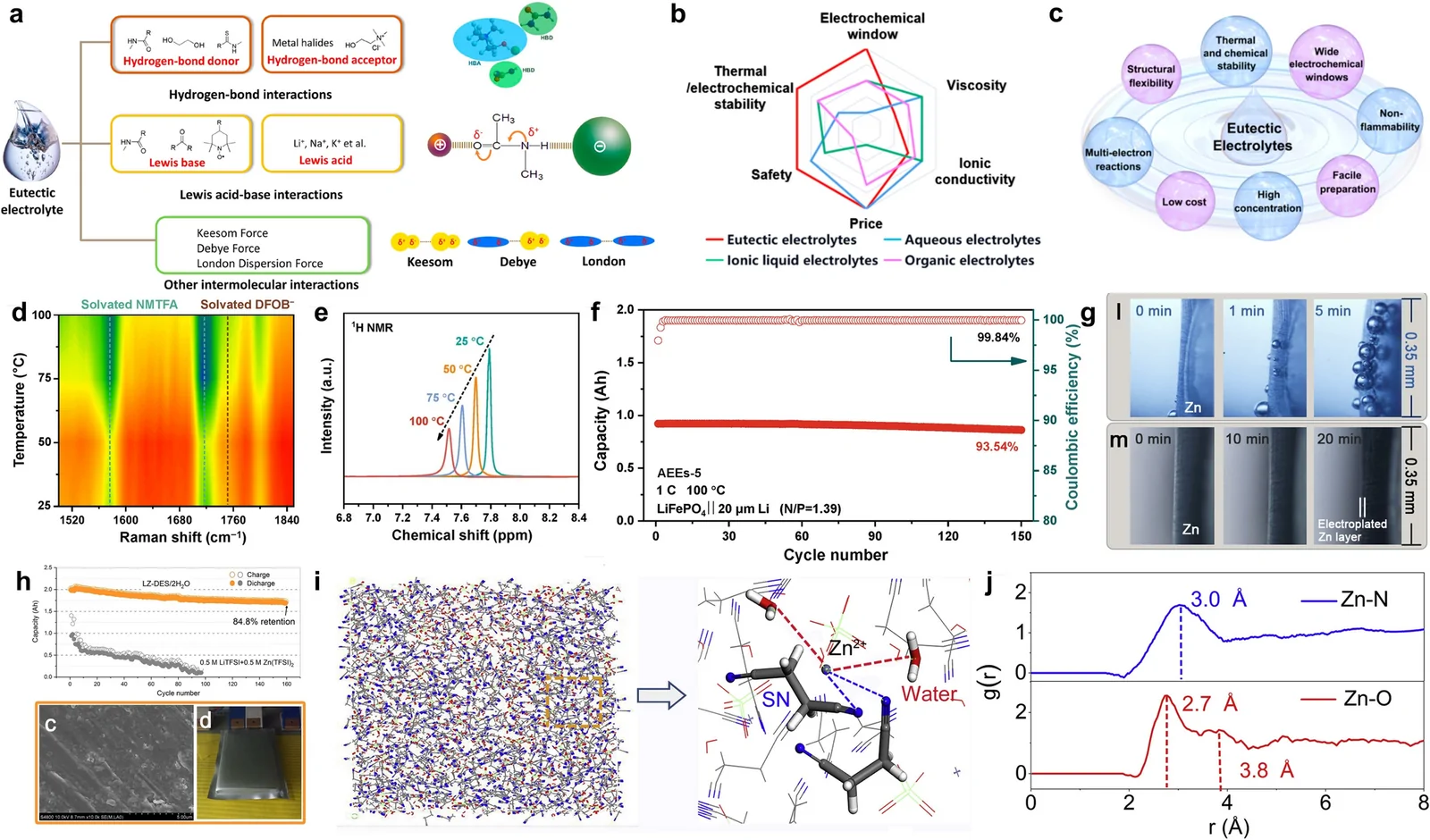
**a** Formation mechanisms of eutectic electrolytes. **a** Reproduced with permission [70]. Copyright 2020, American Chemical Society. **b** Radar plots: the properties of different types of electrolytes. **c** Advantageous properties of eutectic electrolytes. **b,** **c** Reproduced with permission [69]. Copyright 2021, American Chemical Society. **d** Raman spectra of AEEs-5 at various temperatures (the intensity from red [low] to blue [high]). **e** Variable-temperature NMR spectra for varied solvation structure of AEEs-5 during heating: stacked ^1^H NMR. **f** Cycling performance of the LiFePO_4_||Li pouch cell using AEEs-5 at 1 C and 100°C **d–f** Reproduced with permission [76]. Copyright 2024, Cell Press. **g** In-situ optical microscopic images of the Zn electrodeposition process in 0.5 M LiTFSI + 0.5 M Zn(TFSI)_2_ and LZ-DES/2 H_2_O at 0.2 mA cm^−2^. **h** High-capacity pouch-type ZMBs. **g,** **h** Reproduced with permission [77]. Copyright 2019, Elsevier. **i** 3D snapshot obtained by MD simulations and representative Zn^2+^-solvation structure in the ZS electrolyte. **j** RDFs for Zn^2+^-N (SN) and Zn^2+^-O (water) from MD simulations of ZS. **i,** **j** Reproduced with permission [78]. Copyright 2020, Cell Press

Deep eutectic solvents (DESs) are eutectic liquid mixture obtained by mixing at least two components self-associated via intermolecular interaction at a certain molar ratio, and have been popularly known as a new class of ionic liquids (ILs) due to their many similar characteristics, such as low vapor pressure, structural flexibility, nonflammability, good thermal and chemical stability. Massive literature on DES applications has been reported in various fields in recent years, however, the definition of DESs is still a controversial subject because basically the eutectic point exists in all the mixtures of immiscible solid compounds which are able to form intermolecular interaction when put together [68]. Despite DES is hard to distinguish in some binary or ternary mixtures, these liquids are commonly classified in three different types by different intermolecular interactions in terms of electrolyte for electrochemical energy storage applications [69]. As shown in Fig. 15a, Zhang et al. concluded that the formation of eutectic electroly is mainly dominated by hydrogen-bond interactions [70], Lewis acid–base interactions, Keesom forces, Debye forces, and London dispersion forces, the first two of which belong to Coulombic interaction while others belong to van der Waals interactions. On this basis, different from ILs which is mainly composed of discrete anions and cations, almost all molecules in eutectic electrolyte are involved in these different interaction, leading to only complex anions and cations exist in the entire system. This mean that the charge transport in the eutectic electrolyte is predominantly depends on ionic mobility rather than the number of charge carriers, and the ideal viscosity and ionic conductivity can be achieved by adjusting ions with rational size (Fig. 15b). Apart from that, the design of novel eutectic electrolyte system can be also achieved by selecting functionalities, substituents, and/or the composition of mixtures to adjust intermolecular interactions, and/or optimizing the proportion of these componet to a specific mixing ratio. In general, the highly adjustability and designability of DESs coould accommodate some completely different electrochemical systems with attrictive advantages such as wide ESW, low cost, and nonflammability, etc. (Fig. 15c). But the eutectic electrolytes are still in the infancy stage and its enormous potential in electrochemical energy storage remains unexplored. The coordination chemistry of eutectic electrolytes and fundamental understanding of interface chemistry should be devoted to further investigating. In this section, we mainly discuss the effect of special solution structure on physicochemical property of electrolyte and battery performance, as several good reviews have summarized the superior performances and wide application of deep eutectic solvents as electrolytes in the past few years [69–75].

LIBs have always been a popular research area in the field of energy storage with their successfully commercialized example. Therefore, queit a number of the rudiments of new materials are realized in lithium batteries (Table 1). In earlier studies, Hu et al. mixed LiTFSI and acetamide (Ace) at a molar ratio of 1:4 to prepare a room temperature molten salt electrolyte with a eutectic temperature of − 67 °C [79]. Although LiTFSI-Ace eutectic electrolyte has a relatively low ionic conductivity with 1 mS cm^−1^, the electrolyte possesses an expanded ESW of 4.4 V (vs*.* Li/Li^+^). Boisset et al. presented a study on the physical and electrochemical properties of three different DESs based on a series lithium salts (LiTFSI, LiPF_6_ and LiNO_3_) with N-methylacetamide (NMAc) [80, 81]. In these eutectic electrolytes, the interaction of polar groups (–CO–NH–CH_3_) of NMAc with Li^+^ and anions (TFSI^−^, PF_6_^−^ and NO_3_^−^) leads to formation of coordinated complexes which lower the freezing poitns to − 72, − 52, and − 75 °C, respectively. As a result, LiTFSI-NMAc shows best comprehensive performance with ionic conductivity of 1.35 mS cm^−1^ and viscosity of 78.38 mPa s. In fact, the higher polarity of organic ligands can more adequately weaken the electrostatic interaction between cations and anions in salt, which can significantly increase in ionic conductivity values. For example, Cui’s group reported succinonitrile (SN)-based dual-anion DES as a nonflammable electrolyte for Li metal batteries [82]. The prepared SN-based eutectic electrolytes delivered a high ionic conductivity of 2.86 mS cm^−1^ with a large Li^+^ transport number of 0.44 at room temperature, and enabled the Li||Li cell to cycle over 1 year with constant voltage profile. The Raman, FTIR, and NMR measurements were used to optimize Li^+^ solvation structure and detect intermolecular interaction which exist in these systems. As a result, the assemble LiCoO_2_||Li cell exhibit an impressive capacity retention of over 70% for 500 cycles with a cut-off voltage of 3.0–4.7 V. An amide-based eutectic electrolyte (AEEs-5) [73], composed of N-methyl-2,2,2-trifluoroacetamide (NMTFA) and lithium difluoro(oxalato)borate (LiDFOB), enabled high-temperature stable operation of LMBs through thermally regulated molecular motion and dipole orientation of NMTFA. As shown in the variable-temperature Raman spectra (Fig. 15d), the characteristic peaks of the solvated DFOB^−^/NMTFA complexes remained stable between 25 and 100 °C, with an increased proportion of AGG clusters at elevated temperatures, indicating enhanced anion participation. Furthermore, temperature-dependent ^1^H NMR spectra revealed an upfield shift in NMTFA protons (Fig. 15e), supporting the notion that thermal motion weakened the Li^+^-NMTFA interactions. As a result, the assembled 1 Ah LiFePO_4_||Li pouch cell retained 93.54% of its capacity after 150 cycles at 1 C and 100 °C, demonstrating excellent thermal resilience (Fig. 15f).

**Table 1:** Comparison of the different strategies in LIBs

Cathode|anode	Electrolyte	Durability	Oxidative stability	Ionic conductivity	References
Li/Gr	3.6 M LiFSI in DME			7.2 mS cm^−1^	[124]
LiCoO_2/_Li	3 M LiTFSI in triglyme	200@77%	4.5	103 S cm^−1^	[125]
LiCoO_2/_Li	3 M LiTFSI-tetraglyme	200@65%	5	103 S cm^−1^	[125]
Carbon black/Li	LiTFSA:triglyme = 1:5	Over 20	4.7	0.5 S m^−1^	[126]
Carbon black/Li	LiTFSA: tetraglyme = 1:5	Over 20	4.7	0.65 S m^−1^	[126]
Al/Li	1.8 M LiTFSI in EC/DEC (3: 7 by volume)		4.5 on Al		[127]
S/Li	7 M 1,3-dioxolane (DOL): dimethoxyethane(1:1)	100@93.7%		7.9 × 10^–4^ S cm^−1^	[128]
Co-Doped Li_2_O/Li	4 M LiFSA electrolyte in acetonitrile	Over 15			[129]
Li/Gr	4.2 M LiTFSI in AN		> 5 on PT	0.98 mS cm^−1^	[19]
Li/Gr	3.2 mol dm^−3^ LiTFSA/DMSO				[33]
LiCoO_2/_Li	LiTFSA:triglyme = 1:1	100@94.6%	5.5 on Al	1.0 S cm^−1^	[130]
S/Li	LiTFSA:triglyme = 1:1	40@59.1%	5.5 on Al	1.0 S cm^−1^	[130]
S/Li	5 M LiTFSI in DME	200@70%			[131]
S/Li	5 M LiTFSI in DME/1,3-dioxolane	200@60%			[131]
S/Li	5 M LiFSI in DME	800@65%	3.0 on Al		[131]
S/Li	3 M LiFSI in DME	1000@76%			[131]
Li[Ni_0.33_Mn_0.33_Co_0.33_]O_2/_Gr	0.56 mol kg^−1^ LiPF_6_ + 5.5 mol kg^−1^ LiFSi in EA	50@90.3%			[132]
Li[Ni_0.42_Mn_0.42_Co_0.16_]O_2_/Gr	0.56 mol kg^−1^ LiPF_6_ + 5.5 mol kg^−1^ LiFSi in EA	50@67.8%			[132]
LiMn_2_O/Li	6 M LiFSA/acetonitrile	50@94.1%		3.3 mS cm^−1^	[133]
LiMn_2_O/Li	5 M LiFSA/acetonitrile	50@93%		7.1 mS cm^−1^	[133]
LiNi_0.5_Mn_1.5_O_4_/Li	4.27 M LiPF_6_/PC	50@92.3%	5 on Al	0.403 mS cm^−1^	[28]
LiNi_0.5_Mn_1.5_O_4_/Li	4.45 M LiPF_6_/PC	50@85.6%			[28]
Air/Li	3 M LiTFSI in 1,2-dimethoxyethane (DME)				[115]
LiNi_0.5_Mn_1.5_O_4_/Gr	1:1.1 LiFSA/DMC	100@90%	5.5 on Al	1.12 mS cm^−1^	[24]
Na/HC	3.3 M NaFSA/TMP	1000@95		2.2 mS cm^−1^	[12]
Li/Gr	5.3 M LiFSA/TMP	1200@96%			[12]
Na_3_V_2_(PO_4_)_3/_hard carbon	3.3 M NaFSA/TMP	100@93%			[12]
LiNi_0.5_Mn_1.5_O_4_/graphite	5.3 M LiFSA/TMP	100@92%			[12]
LiNi_0.5_Mn_1.5_O_4/_graphite	3.25 M LiFSI-SL	1000@69%	5.5 on Pt	1.1 mS cm^−1^	[134]
LiNi_0.6_Mn_0.2_Co_0.2_O_2/_Li	10 M LiFSI EC/DMC	100@86%	5 on Pt	2 mS cm^−1^	[31]
LiNi_1/3_Mn_1/3_Co_1/3_O_2/_Li	2 M LiTFSI and 2 M LiDFOB in DME	500@80%	4.75 on Pt	2.31 mS cm^−1^	[36]
LiNi_0.5_Mn_1.5_O_4_	7 M LiFSI in FEC	130@78%	5 on Al	1.25 mS cm^−1^	[25]
LiCoO_2_/Gr	2.2 M LiFSI-TEP 5 vol% FEC and 0.05 M LiBOB	50@90%	5.5 on Pt	2.24 mS cm^−1^	[135]
LiFePO_4_/Li		500@80%	5.2 V on Au	2 mS cm^−1^	[136]
(LiNi_0.5_Co_0.2_Mn_0.3_O_2_)/Li	3.5 M LiTFSI-DMC-[C2mpyr] [FSI]	100@95%	5.2 V on Au	2 mS cm^−1^	[136]
LiNi_0.6_Co_0.2_Mn_0.2_O_2/_mesocarbon microbeads	2 M LiTFSI and 2 M lithium difluoro(oxalate) borate (LiDFOB) in trimethyl phosphate (TMP) and gamma-butyrolactone (GBL)	200@83.8%	5.1 on Al		[137]
LiNi_0.8_Co_0.1_Mn_0.1_O_2_ (NCM811)/MCMB	2 M LiTFSI and 2 M lithium difluoro(oxalate) borate (LiDFOB) in trimethyl phosphate (TMP) and gamma-butyrolactone (GBL)	200@76.8%	5.1 on Al		[137]
LiNi_0.8_Co_0.15_Al_0.05_O_2_ (NCA)/MCMB	2 M LiTFSI and 2 M lithium difluoro(oxalate) borate (LiDFOB) in trimethyl	400@81.9%	5.1 on Al		[137]
LCO/Gr	2.0 M LiPF_6_ /DMC: FEC: FEMC (3:1:1 v:v)	500@83.5%	4.5 on Al	9.3 mS cm^−1^	[138]
NCM811/Li	2 M LiFSI/DEE: benzene (PhH) (1:1 v:v)	450@87.3%	4.7 on Al		[139]
NCM811/Li	LiDFOB, LiTFSI, Pyr13TFSI, DME, and TTE were mixed in the molar ratios with 1:4:3.86: 9:15	1800@76.1%	4.5 on Al	4.83 mS cm^−1^	[140]
NCM811/Li	2 M LiFSI /DME:2,2-bis(trifluoromethyl)-1,3-dioxolane (BTFMD) (1:3 v/v)	570@82.2%			[42]
NCM622/Li	LiFSI: LiDFOB: DME: FEC: FB: F3B: TTE = 1:0.05:1.6:0.2:1.87:0.2:0.93	250@75%		6.0 mS cm^−1^	[141]
LiFePO_4_/Li	1.5 wt% PETEA /LiTFSI:2,2,2-trifluoro-N-methylacetamide (1:4)	2500@81.7%			[142]
LiFePO_4_/Li	MEEA (169 uL)、ADN (228 uL)、LiTFSI (197 mg)、PENTA (70 mg)、FEC (21 uL) and AIBN (0.82 mg)	1500@80.78%		2.2 mS cm^−1^	[143]
LiCoO_2_/Li	6 mmol LiBF_4_, 6 mmol LiDFOB,2 mL ethyl 3,3,3-trifluoropropanoate (tFEP), 4 mL FEC	100@93.6%	4.6 on Al	3.03 mS cm^−1^	[50]

Eutectic electrolyte systems are also promising for ZIBs. Cui’s group developed a new water-in-DES electrolyte system by simply adjusting the molar ratio of H_2_O and DES (H_2_O in a eutectic mixture of urea/LiTFSI/Zn(TFSI)_2_), in which the water reactivity preserves a relatively depressed level due to the formation of well-defined eutectic network [77]. As displayed in Fig. 15g, the in-situ optical microscopy observation observation of electrolyte compatibility with Zn show a violent gas evolution occurs in the counterpart eletrolyte, while no gas evolution was observed when the Zn metal was deposited in water-in-DES electrolyte. Moreover, the extended cathodic limit on the metallic Zn substrate implying the hydrogen evolution reaction was greatly inhibited. The high-capacity pouch-type Zn metal batteries were also assmebled to campare the stability with and without DES. Based on the water-in-DES electrolyte, this 2 Ah pouch cell showed high-energy density of 52 Wh kg^−1^ (based on the total device mass) excellent cyclic performance with a capacity retention of 84.8% (after 150 cycles) (Fig. 15h). However, hydration effect contributes a lot to DES clusters, precise control of water content is important the formation of the eutectic network, which lead to a delayed oxidation and suppressed solvating ability. In their following study of Cui’s group, a hydrated eutectic electrolytes Zn(ClO_4_)_2_·6H_2_O/SN for ZIBs with a precise hydration level was reported [78]. As shown in Fig. 15i, the nano solvation structure of Zn^2+^ indicate that both the Lewis basic SN and H_2_O moleculars contributes to the formation of the hydrated eutectic structure, and portion of H_2_O moleculars are mainly sequestered in the outer solvation shell of Zn^2+^ cations. Apart from the peak of the Zn–N pair in 3.0 Å which is identified as SN coordination (Fig. 15j), two peaks of the Zn–O pair can be observed at 2.7 and 3.8 Å, respectively, suggesting that water molecules present in both the primary and second solvation shell of Zn^2+^. Compared with conventional aquause electrolyte Zn(ClO_4_)_2_ in water, the presence of SN in Zn(ClO_4_)_2_·6H_2_O/SN lower the strength between Zn^2+^ and H_2_O, and the eutectic nature of Zn(ClO_4_)_2_·6H_2_O/SN affords rich internal interactions which further saturate the bipolar coordination sites of water molecules. Thus, significantly moderating the parasitic reactions that occurred at the EEI.

## Compatible Rechargeable Batteries

### Metal Anodes

#### Li Metal

Li metal has attracted extensive interest due to its ultrahigh theoretical capacity (3860 mAh g^−1^) and low standard reduction potential (− 3.04 V vs. standard hydrogen electrode). Nevertheless, Li metal is thermodynamically unstable and extremely reactive with traditional organic electrolytes. The side reactions induce the formation of instable and non-uniform SEI film, which simultaneously consume Li metal and electrolyte, thus showing poor CE (< 80%). Another fatal problem is dendritic Li growth on the Li surface. In contrast to carbonate electrolytes, ether electrolytes show relatively better compatibility with Li metal because of good cathodic stability of ether solvents. Ether molecules, however, are unstable beyond 4.0 V (vs. Li^+^/Li) and cannot be utilized in high-voltage cathodes. DMSO-based electrolytes tend to severely exhaust the Li anodes with ultralow Coulombic efficiency, which is ascribed to the direct reduction of the Li^+^, DMSO complex. Moreover, the physical features of SEI could significantly affect the nucleation and growth of Li dendrites, inducing different morphological Li dendrites, such as mossy, whisker, and nodule-like Li. Therefore, extensive researches have been conducted to develop stable organic electrolytes, especially fluorinated SEI, against Li metal for favorable Li-deposition behaviors and long-term cycling stability (Table 1) [83].

HCEs are proven to promote stable lithium anode cycling. They exhibit effective suppression of lithium dendrite growth and shape change in the metallic lithium anode. Zhang’s group have done systematic investigation on this direction [84]. They reported a HCEs consisted of 4 M lithium bis(fluorosulfonyl)imide (LiFSI or LiN(SO_2_F)_2_) in DME. Needle-like dendritic Li deposition occurred in a 1 M LiPF_6_-PC electrolyte. In contrast, favorable Li plating was observed in the concentrated 4 M LiFSI-DME electrolyte, in which nodule-like Li with round-shaped edges is formed. Unlike the nano-needles formed in dilute electrolyte, the Li nodules are in much large diameters on the order of ~ 10 µm that decrease the likelihood of penetrating the separators. Moreover, the deposited surface film composed of Li nodules showed much lower surface are than that of the Li nanoneedles. Accordingly, the side reductions between the plating Li and electrolyte are much less in the HCEs than in 1 M carbonate-based electrolyte, leading to greatly enhanced CE (99.2%) during Li stripping/plating process. In spite of enhanced stability of Li metal, such ether-based HCEs enable stable battery cycling at high voltage (4.5 V). According to DFT calculaitons, with the increasing molar ratio of LiFSI to DME, the HOMO energy levels of the solvation complexes of LiFSI-DME shifted to lower values due to the donation of the lone electrons of oxygen atoms to Li cations in the solvation complexes. The corresponding cylic voltagmmetry curves (CVs) indicated that the concentrated LiFSI-2.0DME (i.e., 3 M LiFSI in DME) electrolyte exhibited highly reversible deintercalation/intercalation processes against electrochemical oxidation. LiFSI-2.0DME is believed to induce an effective passivation mechanism to avoid the ether decomposition on the electrode surface.

Beyond ether-based systems, carbonate-based HCEs were developed by dissolving LiFSI in DMC. Increasing the LiFSI concentration from 2 to 6 M enhances the CE of Li plating/stripping from 20% to 98.7%. A maximum CE of 99.3% is achieved at 10 M, with excellent cycling stability over 1000 cycles. The LiF-rich SEI formed predominantly from LiFSI reduction plays a critical role in suppressing dendrite growth and minimizing electrolyte consumption. XPS confirms the significantly different SEI compositions in dilute and concentrated electrolytes: the SEI formed in HCEs contains much higher F and S content, but lower C and O content, than that formed in carbonate-based dilute electrolytes. The abundant LiF originates from the reduction of FSI^−^ rather than solvent molecules. This unique interphase is crucial for stabilized Li deposition/stripping: LiF, being an electronic insulator, effectively prevents electron leakage through the interphase, thereby suppressing continuous electrolyte decomposition and inhibiting dendrite formation. Meanwhile, the high interfacial energy between LiF and Li facilitates rapid and uniform Li^+^ transport along the interface, promoting homogeneous Li deposition and further mitigating dendrite growth [83].

#### Na Metal

Similarly, Na anodes are highly reactive toward organic electrolytes as well, which need to be stabilized to allow reversible Na cycling via passivating SEI. Various HCEs, such as 4 M NaTFS/TEGDME [85], enable reversible plating/stripping of solid Na metal with an extremely high Coulombic efficiency. A highly concentrated ether-NaFSI solution (2.5 M NaFSI-DME) was reported, which largely passivated the Na metal surface and minimized side degradation reactions during cycling [86]. Freunberger and co-workers reported a concentrated electrolyte with a NaFSI/DME molar ratio of 0.5 that allowed nondentritic Na metal cycling with ~ 97.7% coulombic efficiency for up to 300 cycles [87]. The unique SEI film is formed due to the decomposition of FSI anions [88]. Lee et al. further optimized this electrolyte system to an ultra-concentrated electrolyte consisted of 5 M NaFSI in DME. Using this electrolyte, a very high CE (99.3%) was achieved for Na plating/stripping over 120 cycles. Meanwhile, unlike the dilute electrolyte, the ultra-concentrated electrolyte could completely avoid corrosion of the Al cathode current collector. The surface layer formed on the electrode is one of the key factors that affect the kinetics and reversibility of the Na plating/stripping process. An unstable SEI deteriorates the plating/stripping kinetics of Na metal. Nonuniform Na metal plating was observed in dilute 1 M NaPF_6_-EC/PC, without showing characteristic silver color of Na metal on Cu current collector. Na metal plating occurred near the center of the Cu substrate in 1 M NaFSI-DME, dark-brown-colored by products due to electrolyte decomposition during Na plating were located at the edge of the substrate. By contrast, uniform and silver-color Na metal plating was observed across the entire surface of the Cu. Overall, the Na cells resulted in substantially improved rate capability and outstanding cycling stability for plating/stripping in the 5 M NaFSI-DME [89]. DMSO is a promising solvent for M-O_2_ batteries. Wu and co-workers reported the enhanced stability of Na metal containing (> 3 mol kg^−1^) NaTFSI in DMSO. Na stability in DMSO is visually investigated via putting Na pieces into O_2_-saturated NaTFSI/DMSO solutions with varied concentrations. Impressively, Na metal showed slight reaction with DMSO solvents, leading to yellowish color after one week in 2.7 and 3.2 M NaTFSI/DMSO solutions. By contrast, Na metal in the 4.1 M NaTFSI/DMSO solution is highly stable with clear solution after one week. Moreover, the CV curves suggested reversible Na deposition/dissolution in the 3.2 M NaTFSI/DMSO solution but not in the dilute 0.9 M NaTFSI/DMSO, confirming improved Na stability in concentrated solutions [90].

#### Zn Metal

ZIBs are highly attractive for large-scale energy storage applications owing to their intrinsic safety, environmental benignity, and low cost. However, their practical deployment remains hindered by severe dendrite growth and parasitic side reactions. Regulating the Zn^2+^ solvation structure has emerged as an effective strategy to enhance interfacial stability. In conventional dilute electrolytes, Zn^2+^ is strongly coordinated with H_2_O to form the [Zn(H_2_O)_6_]^2+^ complex, resulting in a high desolvation energy barrier, nonuniform Zn^2+^ flux, and subsequent dendrite formation. Shi et al. designed a WSEs by introducing butanone as an additive to modulate the Zn^2+^ solvation structure [91]. Raman, FT-IR, and ^1^H NMR analyses confirmed the solvation transformation: the strong H-bonding between ketone molecules and water decreased the intrinsic solvation ability of H_2_O, thereby weakening Zn^2+^-H_2_O coordination. The average coordination number of H₂O in the Zn^2+^ solvation sheath decreased from 5.6 to 4.3, while SO_4_^2−^ coordination increased to 1.3, indicating a structural evolution from “SSIPs” to coexisting “SSIPs + CIPs”. The increased anion participation accelerated Zn^2+^ desolvation kinetics through electrostatic repulsion at the electrode surface and suppressed the release of reactive water molecules, fundamentally mitigating electrode failure. Consequently, an NVO cathode achieved 99.1% capacity retention after 20,000 cycles at 5 A g^−1^.

Side reactions in ZIBs are primarily driven by heterogeneous Zn deposition and inevitable water decomposition. Recently, an interphase-enhanced localized high-concentration electrolyte (ILHCE) was reported [92], where π–H–H interactions between emim^+^ and 1,4-dioxane drew diluent molecules into the electrical double layer and formed an anion-rich, water-poor structure. This optimized Zn^2+^ solvation into [Zn(H_2_O)_4_(OTf)_2_], lowered the desolvation barrier, suppressed dendrite growth, and reduced water participation in hydrogen evolution. As a result, Mn_0.5_V_6_O_13_-based full cells retained 80% of their capacity after 300 cycles. Huang et al. further proposed an “anion–diluent matrix (ADM)” strategy using amphoteric 2,2,3,3-tetrafluoro-1-propanol (TFP) as an anion-affinitive diluent to construct an ion-decoupled localized high-concentration electrolyte (ID-LHCE) [93]. TFP formed an ADM through dual-site H-bonding with OTF^−^, which disrupted the continuous water network and confined water molecules within isolated water-rich nanodomains. This reduced water activity and suppressed hydrogen evolution and corrosion. At the same time, the ADM released ~ 60% of OTF^−^ from the Zn^2+^ solvation sheath, increased the Zn^2+^ transference number to 0.72, and decreased the desolvation barrier, thereby inducing dense, hexagonally layered Zn deposition and eliminating dendrite-triggered micro-short circuits. The Zn||NaV_3_O_8_·1.5H_2_O full cell delivered 72.5% capacity retention over 2000 cycles, and a 1.04 Ah pouch cell with a high areal loading of 15.29 mg cm^−2^ operated stably, confirming the practical viability of this strategy.

### Intercalation-type Anodes

#### Graphite Anode

For next-generation Li-ion batteries, even though high-capacity silicon-based materials stands out due to its high theoretical capacity (~ 4200 mAh g^−1^) and favorable charge voltage (~ 0.3 V vs. Li^+^/Li), which are already partially commercially available and are expected to serve as a mainstream anode in the future. The research on anodes has not obtained dramatic breakthroughs yet. Graphite is still dominant as active anode materials. However, the organic electrolyte is limited to be EC-based. Yamada’s group found that the graphite anode exhibited totally different behavior in a HCEs. They believed that the HCEs with inert PC or DMSO solvents can suppress the cointercalation of solvents and allow for reversible Li intercalation into the interlayer of the graphite [27, 94].

#### Hard Carbon

Wang et al. demonstrated excellent cyclability of hard carbon by simply increasing the concentration of NaN(SO_2_F)_2_ (NaFSA) to 3.3 M in a flame-retardant trimethyl phosphate (TMP) solvent. The selection of NaFSA salt is due to its weak cation–anion interaction, ahieving high ion transport even at high concentrations. TMP solvent has extinctively features in terms of high oxidative stability and low viscosity. It is intersting hard carbon cannot normally work in dilute 1.0 M NaFSA/TMP electrolyte. In contrast, the hard carbon showed much enhanced Coulombic efficient of 75% with remarkably suppressed electrolyte domposition in concentrated 3.0 M NaFSA/TMP electrolyte, which indicated very different passivation chemistry between two electrolytes. X-ray photoelectron spectroscopy (XPS) analysis revealed that the solid-electrolyte interphase on the hard carbon surface was mainly composed of F, S, N, Na, and O elements. They deduced that the SEI growth in concentrated electrolyte is based on the reduction of FSA^−^ anions, which is prone to produce a uniform passivation film, thus leading to a reversible battery performance over 1500 cycles at 5 C. On the other hand, another positive feature of this SEI film is high reaction kinetics due to fast Na^+^ transport at the EEI. However, the reduction of TMP solvents was domient in the 1.0 M electrolyte, which resulted in the formation of large particles rather than a compact uniform SEI film, unable to passivate the hard-carbon anode [12].

### High Concnetration Electrolytes for Cathodes

#### High-Voltage 5 V Cathodes

In order to achieve high energy, an ideal cathode is supposed to possess high capacity as well as high discharge voltage. A range of cathode materials have been explored, including layer-structured mixed metal oxides (LiNi_1/3_Mn_1/3_Co_1/3_O_2_) [95], various polyanionic compounds (LiMPO_4_, M = Fe, Mn, Co, or Ni) [96], high-voltage spinel (LiNi_0.5_Mn_1.5_O_4_) [97], and Prussian blue analogues (PBAs) [98]. By contrast to the active research on electrode materials and battery systems, the improvement on electrolytes is very limited in the past decade. Electrolytes serve as an indispensable component in rechargeable lithium and beyond-lithium batteries. Typically, electrolytes are consisted of alkaline metal salts and organic solvents, which not only deliver fast alkaline metal ions flow between the cathodes and anodes but also form passivation films on the EEI. The conventional lithium-ion electrolyte is a solution by dissolving 1 M LiPF_6_ in an EC-based solvent. In this system, the optimized concentration is 1 M due to the highest Li-ion conductivity. The selection of EC is imperative for the commercial graphite anode, which can decompose on the graphite surface and kinetically stabilize graphite anode by forming SEI. The stability and robustness of SEI is very critical that could effectively terminate continuous electrolyte decomposition on the fresh electrode. Some reducible and oxidizable additives can be applied into the carbonate electrolytes, so as to form protective films on the electrodes and stabilize the electrode-interface. This electrolyte formula has been in use since the commercialization of lithium-ion batteries. The existing industry infrastructures are established for producing LiPF_6_ and organic carbonates, which can satisfy the requirements for most of LIBs and even Some high-voltage cathodes, such as Li_2_MnO_3_ (4.5 V) [99], LiNi_0.5_Mn_1.5_O_4_ (4.6 V) or LiCoPO_4_ (4.8 V), however, operate at high voltages which are above the stability limit (4.3 V) but suffers from oxidative decomposition of carbonate solvent. Sodium-ion batteries, which normally show ~ 0.3 V lower operating voltage. Similarly, sodium- and potassium-ion batteries follow the research patterns on seeking for various cathode electrode materials beyond LIBs. Thus, with the expectation of high-energy batteries, new bulk electrolyte solvents are urgently needed for high-voltage LIBs, SIBs and KIBs. On the other hand, the organic electrolytes, especially the carbonate-based electrolytes, are highly flammable, leading to fires or even explosions.

Yamada’s group reported an effective HCEs for LiNi_0.5_Mn_1.5_O_4_ electrode-based 5 V LIBs [39]. Lithium bis(fluorosulfonyl)amide (LIFSA, LiN(SO_2_F)_2_) salt is selected due to its modest dissociative energy and stability; oxidation-stable dimethyl carbonate is favorable as the solvent. The LiNi_0.5_Mn_1.5_O_4_ cathode was failed in dilute 1:10.8 LiFSA/DMC electrolyte due to the continuous Al dissolution at 4.3 V. This parasitic Al dissolution remained when enhancing the concentration to 1:1.9, with possible cycling up to the cut-off voltage of 4.9 V. In contrast, the concentrated 1:1.1 LiFSA/DMC electrolyte rendered reversible lithiation/delithiation process at a high voltage of 5.2 V. With an effective inhibition of anodic Al dissolution, the LiNi_0.5_Mn_1.5_O_4_ cathode showed much enhanced cycling stability with reversible capacity of ~ 125 mAh g^−1^ over 100 cycles. The assembled LiNi_0.5_Mn_1.5_O_4_/graphite 5 V-class battery delivered prolonged cycling stability, excellent rate capability and high safety in HCEs than a commercial electrolyte. The improvement is attributed to the following reasons: (i) The HCEs is believed to greatly restrain the transition-metal dissolution of the cathode and suppress the anodic Al dissolution; (ii) concentrated electrolytes can form anion-derived SEI film on the negative electrode, which can successfully passivate the graphite surface and result in low interfacial resistance with rapid Li^+^ intercalation kinetics.

#### ***O***_***2***_*** Cathodes***

The commercialization of metal-O_2_ batteries has been largely impeded by the decomposition of organic electrolyte [100, 101]. For Li-O_2_ batteries, the classic reaction pathway is based on two tandem one-electron steps with superoxide as the sole product, namely O_2_ → O_2_^−^/LiO_2_ → Li_2_O_2_, and the oxygen evolution reaction is the direct oxidization of Li_2_O_2_ to O_2_ [102–104]. The O_2_^−^/LiO_2_ intermediate is highly nucleophilic, which enforces the electrolytes shift from conventional carbonates [105] to strong Lewis basic solvents such as ethers [106, 107], nitriles [108], and sulfones [109]. A Na-O_2_ battery shared similar mechanisms with its Li-O_2_ cousin [101]. Both peroxide (Na_2_O_2_) and superoxide (NaO_2_) are possible final products [110]. Different reaction pathway can be affected by different selection of carbon-based cathode materials, catalysts, and electrolyte [111]. The high reactivity of peroxide and superoxide in Li-O_2_ and Na-O_2_ batteries directly leads to parasitic reactions with electrolytes [112]. It is obvious that the stability of electrolyte toward reduced oxygen intermediates and final products plays a crucial role in M-O_2_ batteries.

HECs have been summarized to greatly stabilize Li metal anodes benefit by the fundamental nature of salt-solvent coordinated solution structures [113]. Liu et al. found that the high-concentrated LiTFSI/TEGDME electrolytes (2-3 M) enable the Li-O_2_ batteries much higher capacity than low concentrated electrolytes (1 M or less). They believed that the growth of Li_2_O_2_ discharge product could be adjusted by tailoring the Li^+^ concentration in the electrolytes [114]. Zhang’s group tested Li-O_2_ batteries in LiTFSI/DME electrolytes with three salt concentrations (1, 2, and 3 M) When tested at 0.1 mA cm^−2^ under a capacity-limited (1000 mAh g^−1^) protocol, the batteries in 1 and 2 M electrolytes showed large increase in overvoltage and significant decay in capacity during cycling [115]. In contrast, the Li-O_2_ cells in 3 M electrolyte maintained very stable discharge capacity at 1000 mAh g^−1^ over 55 cycles. DFT calculation indicated that all the DME solvent molecules were coordinated with salt cations, thus mitigating the decomposition of the HCEs due to difficult C–H bond scission of the DME molecules. The enhanced stability in HCEs during discharge/charge cycling benefited from both a better Li anode and reversible O_2_ cathode. Li metal is more stable in concentrated electrolytes due to the stable and high-conductivity SEI layer formed on the Li-metal anode. On the other hand, the intermediate in O_2_ electrodes are more ionically conductive. Using a concentrated NaTFSI/DMSO (> 3 mol kg^−1^) electrolyte, Na–O_2_ batteries can achieve enhanced cycling lifespan with NaO_2_ as the sole discharge product, highlighting the superior properties of HCEs for M–O_2_ batteries [90].

#### Sulfur Cathodes

In addition to the cathode design, the electrolyte plays a more crucial role in M–S batteries system, which is due to that the solubility of polysulfide intermediate depends on the solvents. Organic solvent with high donor numbers preferentially coordinate with Lewis acidic cations such as Li^+^, which show strong ability to dissociate the salts resulting in high ionic conductivity. When adding a high concentration of Li salts into ether solvents, the obtained solution can decrease the ability to solvate other lithium species, this is, lithium polysulfides. This concept was first explored in a TEGDME-Li equimolar complex [116, 117]. Similar solvation properties were found in a highly concentrated 7 M LiTFSI-DOL: DME electrolyte, which showed a very low solubility for Li_2_S_8_ while simultaneously demonstrating comparatively sustainable cycling with high capacity retention [118]. Dokko et al. examined the Li/S performance by increasing the LiTFSI concentration in a TEGDME solvent, which also proven that the solvate ionic liquids can greatly suppress the polysulfide solubility, leading to high reversible capacity, prolonged cycling life as well as high Coulombic efficiency. Via driving the electrolyte system into a solvate regime, all of the solvent is subject to strong interactions with the LiTFSI results in only small measured solubility of polysulfides [119]. Nazar’s group investigated a solvent-salt complex of acetonitrile (ACN)_2_-LiTFSI as a new class of Li–S battery electrolyte, in which all CAN molecules are bound by complexation, thus leading to suppressed electrolyte reaction with lithium metal and LiPS solubility The very limited dissolution and mobility of LiPS strongly affect the speciation and polysulfide equilibria, leading to enhanced capacity and controlled formation of Li_2_S [120]. However, Li metal is extremely intrinsic instable in ACN. The existence of certain free ACN solvent requires large excess Li metal to achieve a sustainable cycling, which largely reduced the energy density of Li–S batteries. Recently, Wang and co-workers formulated an ultra-concentrated electrolyte by dissolving 12 M LiFSI in dimethoxylether (DME). The polysulfide dissolution and migration are not only efficiently inhibited but also the irreversible reaction between Li metal and ether solvent is minimized. Meanwhile, the LiF-rich interphase from the FSI^−^ anion can effectively supresses the formation of Li dendrites. The CV comparison suggested that at direct and deep reduction of solid S_8_ to solid-state Li_2_S in concentrated electrolyte, which is very distinct mechanism in conventional electrolyte from sulfur to high-order polysulfide and to low-order polysulfide. The difference in electrochemical behaviors is ascribed to the changes in competitive solvation of Li^+^ by solvent molecules and FSI^−^ anions. The cells showed high cycling stability and Coulombic efficient over 300 cycles, maintaining capacities of 786 and 644 mAh g^−1^ at 0.1 and 1 A g^−1^, respectively [121]. Recent systematic studies by Li et al. on WSEs further deepened the mechanistic understanding of solvation-structure regulation in electrolyte systems [122]. Using DOL and DME as the base solvents, a series of weakly solvating solvents (WSSs), including hexyl methyl ether (HME), diisopropyl ether (DIPE), and diisopropyl sulfide (DIPS), were introduced to construct WSEs. The ESP was found to exhibit a two-stage variation with increasing WSS content, where the critical transition point depended on whether the WSS molecules entered the inner solvation shell of lithium polysulfides (LiPSs). When the WSS content was below 20%, the WSS molecules remained confined to the outer solvation shell, resulting in a relatively moderate change in ESP. However, once the WSS content exceeded this threshold, the WSS molecules directly coordinated with LiPSs, leading to a sharp decrease in ESP. This coordination also markedly retarded the charge-transfer kinetics of LiPSs and significantly increased activation polarization and activation energy. To address the deteriorated reaction kinetics while retaining the intrinsic advantages of WSEs in suppressing LiPS shuttle and protecting the Li metal anode, a titanium nitride (TiN) electrocatalyst was introduced to regulate the energy states and chemical environment of LiPSs at the cathode–electrolyte interface. This catalytic regulation effectively reduced activation polarization and restored interfacial reaction kinetics, while maintaining the low-solubility benefits afforded by the WSEs. Benefiting from this synergistic strategy, a 10 Ah Li–S pouch cell achieved a practical energy density of 607 Wh kg^−1^ and demonstrated stable cycling over 17 cycles.

In HCEs, the polysulfide dissolution for both Li–S and Na–S batteries can be extremely inhibited, which arised from the strong complexation of solvent molecules with alkili metal cations, remaining very little free solvent availble for polysulfide sovation [123].

### Others

DESs have also been used to test as electrolyte for other energy storage applications, such as alumium batteries, supercapacitors and redox flow batteries. These various system have very different requirements for electrolyte. Such as redox flow batteries which require one component should have high redox activity, while another component should act as the supporting salt. This is quite distinct from metal-based rechargeable batteries which should maintain their chemical stability during charging/discharging. Another example, rechargeable Al ion batteries involving a multi-electron redox reaction are attractive for the development of next-generation rechargeable battery systems. However, the multivalent reaction occurs in electrolytes, electrodes and EEI caused a series of problem such sluggish dynamics and strong desolvation activation energy, which hinder the development of Al ion batteries. Al^3+^ with a small ionic radius of 0.0535 nm (Li^+^ with an ionic radius of 0.076 nm) but higher surface charge density [144]. This means that Al-based salt with a much stronger Coulombic interaction between cations and anions, which is hard to be dissolved in commonly used organic solvents, leading a to low ion concentration and ionic conductivity of the electrolyte. DESs may give an acceptable solubility of the regular Al-based salt AlCl_3_ by forming AlCl_4_^−^ anions and [AlCl_2_·(ligand)_n_]^+^ cations through the heterolytic cleavage of AlCl_3_, mean that the traditional solvent–solute ionization strategy is no longer applicable. Overall, the solvent structure of DESs needs to be further explored and analyzed in the research of these batteries system, because there are rare studies have been reported in this regard.

## Promising Improvement for Electrolyte Design

### Advanced Characterization Techniques

Systematically characterizing the solvation structure of ions and EEI structure/composition are crucial to directly and accurately obtain the actual properties of electrolytes and interfaces, which are closely related to the overall performance of electrochemical devices. Conventional characterization techniques for solvation structure including NMR, Raman spectroscopy, FTIR, X-ray absorption fine structure (XAFS) spectroscopy, extended X-ray absorption fine structure (EXAFS) spectroscopy, small-angle X-ray scattering (SAXS) and so on. And the characterization techniques for EEI including electron microscopes, XPS, atomic force microscope (AFM), electrochemical quartz crystal microbalance (EQCM), time-of-fight secondary-ion mass spectrometry (TOF-SIMS) and so on. However, it is difficult to in-situ and operando monitor the electrolyte composition within full cells. Miele et al. reported an operando Raman spectroscopy method by embedding a hollow-core optical fiber probe inside a pouch cell, which reveals changes of the components of the electrolyte and show the potential to track the cation solvation dynamics [145]. Wang et al. used in-situ Raman spectroscopy to investigare the structure and dynamic process of water at the solid–liquid interface, which reveal interfacial water consists of hydrogen-bonded and hydrated Na^+^ water [146].

### Advanced Computational Simulation

As a powerful tool to visualize the solvation structure of electrolyte, MD simulations (including classical, Ab Initio, and machine-learning molecular dynamics simulations) are widely applied in research on various electrolyte systems. Typical applications including cation/anion solvation structures, electrode–electrolyte interfacial structure, prediction of electrolyte properties, ionic-transport mechanisms, etc. These calculations help deepen the understanding of practical electrolytes at the atomic-level or molecular-level, which are differcult to reveal by conventional characterizations methods such as FTIR, Raman and NMR spectrum. Among the three methods mentioned above, classical and Ab Initio MD simulations are the most commonly used methodology, which are almost the standard configurations in majory electrolyte reseaerches. The collective behavior of the whole electrolyte system can be analyzed through the calculation of the interaction forces and related statistical analyses. Notably, the selection of the force field should be carefully considered because their different optimized timescales and numbers of atoms, which significantly influence the accuracy [147]. However, the empirical force fields of classical MD simulation suffer from the imponderable simulation error and difficult parametrization, and the unaffordable simulation costs of Ab Initio MD simulations is not expected to be used in large-scale application. Machine-learning as a emerging technology has brought tremendous new opportunities for both theoretical and experimental studies in many fields [148]. For example, the machine-learning MD simulations of 10- and 100-million atoms have been achieved through a deep-learning potential approach in the water and copper systems, respectively, which is considered as an extremely important breakthrough in the field of MD simulations [149, 150]. For electrolyte design, machine-learning MD simulations are expected to deal with a large-scale simulation of electrolytes system and even complicated electrode–electrolyte interfaces in the future. These simulations and computational results will be profitable for us to establish more profound comprehension about the structure and properties of battery system.

## Conclusions and Outlook

**Fig. 16:**
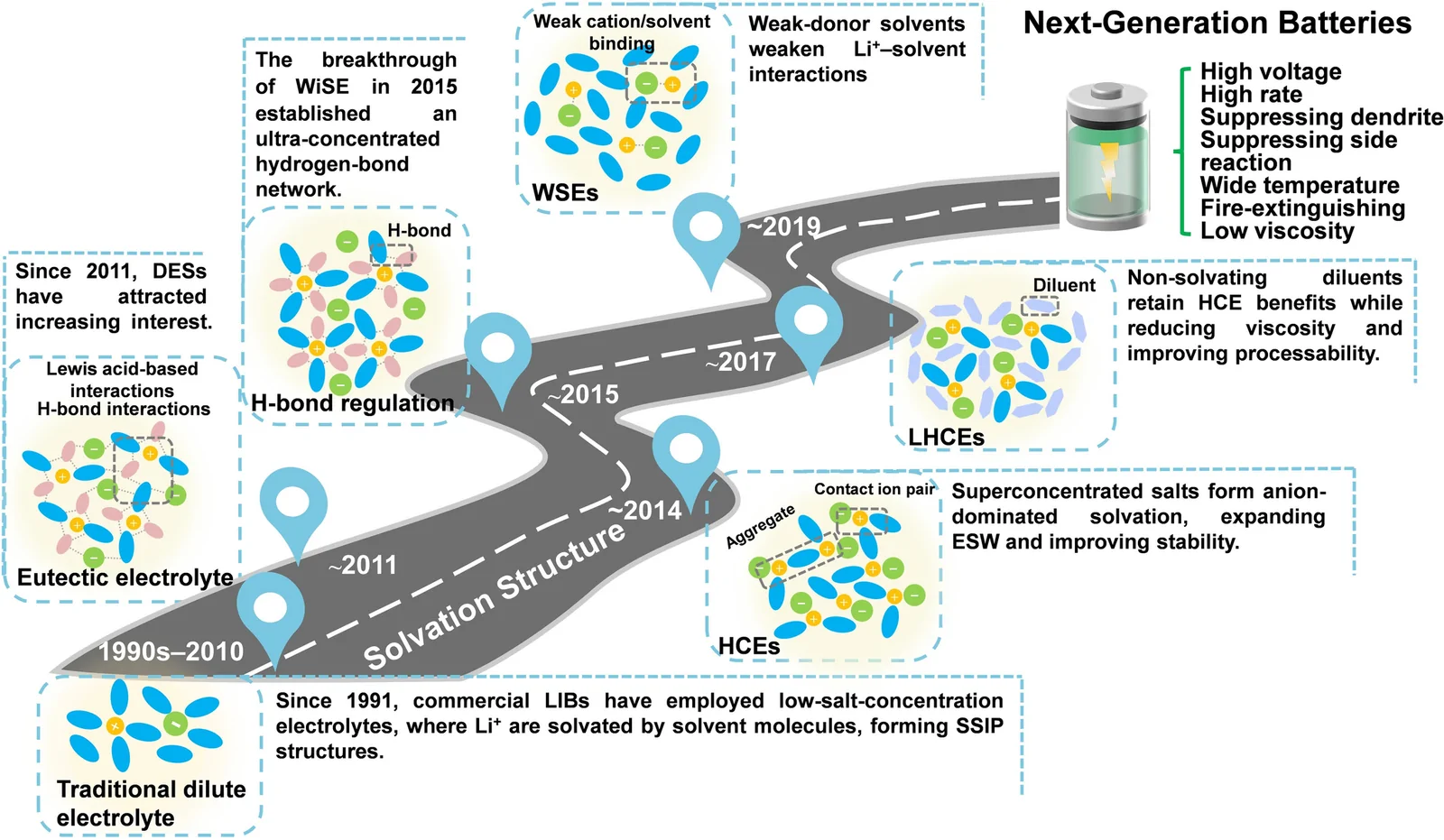
Evolution roadmap of electrolyte solvation structures for next-generation batteries

Against the background of global decarbonization and carbon neutrality, rechargeable batteries have become a key solution to address the intermittency and instability of renewable energy. This review has highlighted electrolyte design strategies that regulate solvation structures, including highly concentrated electrolytes, localized high-concentration electrolytes, weakly solvating electrolytes, hydrogen-bond regulated electrolytes, and eutectic electrolytes (Table 2). By optimizing ion–solvent interactions, enabling robust SEI formation, and widening the ESW, these strategies significantly enhance cycling stability, safety, energy density, and operating temperature range. For instance, HCEs minimize free solvent molecules and shift the LUMO to the anion, expanding the ESW of aqueous electrolytes from 1.23 V to beyond 3.0 V, thereby enabling advances in diverse systems such as Li, Na, Zn ion batteries, as well as Li–S, Li–air, and Na–S batteries. Furthermore, derivative strategies such as LHCEs mitigate the drawbacks of HCEs, including viscosity and cost, while delivering superior performance in high voltage (> 5 V) and fast-charging applications. In addition, the development of novel solid-state electrolytes warrants special attention. A case in point is the solid-state solvation structure recently proposed by Zhang’s group [151], which utilizes a solid-solution method to construct a cation solvent aggregate. This design has enabled all-solid-state batteries to achieve both high operating voltage and long-term cycling stability. Inspired by this approach, the future design of solid electrolytes can further draw upon principles from liquid electrolytes, potentially advancing through the optimization of cation coordination environments and overall composition. Collectively, these electrolyte engineering approaches overcome the inherent limitations of conventional dilute electrolytes, such as narrow ESW, flammability, and low-temperature failure, thus providing a solid foundation for the development of sustainable and high-performance energy storage devices. Through systematic literature analysis, this work emphasizes the central role of solvation-structure design in understanding ion transport, interfacial processes, and overall battery performance, thereby establishing both theoretical and practical frameworks for next-generation electrochemical energy storage technologies (Fig. 16).

**Table 2:** Comparison of Trade-off Metrics for Different Electrolyte Strategies

Strategy/Metric	HCEs	LHCEs	WSEs	H-bond regulation	Eutectic electrolyte
Salt concentration	Very high	High	Medium	Medium	Medium–High
Viscosity	Very high	Moderate	Low	Variable	Medium–High
Ionic conductivity	Low–Moderate	Moderate	Moderate	Variable / Moderate	Moderate
Cost	Very high	High	High	Medium	Medium
ESW	High	High	Moderate	Moderate	Moderate
Anode compatibility	Good	Good	Moderate–Good	Good	Moderate
Cathode compatibility	Good	Good	Moderate	Moderate	Moderate–Good
Safety	Medium	Medium–High	Medium	Medium–High	High
Manufacturability	Poor–Low	Good	Good	Good	Moderate
Long-term stability	High	High	Medium	Medium	Medium–High
Typical application scenarios	LMBs, Li–S, High-voltage Li systems	LMBs, High Voltage, High-rate capability,	Li–S, High-rate capability, Low temperature	Aqueous systems, Low temperature	Specialty Zn and Li systems, Non-flammable, High-temperature

Despite these promising advances, several scientific and technical challenges remain. A fundamental issue lies in the precise characterization and visualization of solvation structures. Although advanced spectroscopic techniques (e.g., Raman, FTIR, NMR) can infer solvation environments through changes in vibrational spectra, the direct correlation between specific ionic solvation structures and macroscopic battery performance is often ambiguous and subject to debate. The transparent, colorless nature of liquid electrolytes also makes them difficult to image directly, unlike solid electrode materials which can be readily examined via SEM or TEM. Nevertheless, the performance enhancements resulting from optimized solvation structures are undeniable, underscoring the need for more sophisticated in-situ/operando analytical tools and computational modeling to bridge this understanding gap.

From a manufacturing standpoint, electrolytes with unique solvation structures (e.g., HCEs, LHCEs) often face poor processability due to high viscosity, which leads to inadequate wettability of separators and thick electrodes, ultimately impairing cell performance-particularly under low temperatures where ionic conductivity drops significantly. Future efforts should focus on optimizing physical properties such as viscosity and conductivity without compromising solvation-structure benefits. This may involve developing novel diluents (e.g., hydrofluorinated ethers) or alternative solvent systems to improve rate capability and low-temperature operation.

Looking forward, the following aspects will be critical for the translation of solvation-structure-engineered electrolytes into commercial next-generation batteries:

1. *Comprehensive Performance and Cost Analysis* A rigorous, standardized evaluation framework is urgently needed to compare the merits and limitations of various advanced electrolytes. This analysis should extend beyond basic electrochemical performance metrics (e.g., Coulombic efficiency, cycle life, rate capability). This involves not only the raw material cost of salts, solvents, and additives but also processing costs influenced by viscosity (e.g., required drying time) and stability. The cost-performance benefit must be justified for target applications, from consumer electronics to grid storage. The synthetic complexity, availability of precursor materials and environmental regulations governing their production must be evaluated for gigawatt-hour-scale manufacturing. Compatibility with existing electrode architectures (e.g., thick electrodes for high-energy density) and roll-to-roll coating processes is crucial. Specifically, poor electrolyte–electrode compatibility can significantly affect both battery performance and manufacturability. Insufficient wetting between the electrolyte and the electrode may create “dry zones” within the electrode interior, leading to increased local impedance and limited active-material utilization, thereby reducing practical capacity and rate capability. In addition, the electrolyte must remain chemically compatible with polymeric binders to avoid swelling or decomposition that could disrupt the mechanical integrity of the electrode. Poor compatibility may further necessitate additional process modifications (e.g., adjustments in coating speed, temperature, or pressure) or even equipment upgrades, which substantially increases manufacturing complexity and cost.
2. *Advanced In-Situ Characterization Techniques* Current understanding of electrolyte solvation structures remains constrained by the limited spatiotemporal resolution and interfacial sensitivity of existing characterization methods. Conventional techniques, such as standard spectroscopic analyses and computational simulations, mainly provided bulk-averaged and static information, which made it difficult to capture the dynamic evolution of ion coordination environments and transient intermediates during cycling. Moreover, these techniques lack the ability to resolve structural and chemical changes occurring specifically at the nanometer-scale electrode–electrolyte interface. Future breakthroughs will rely on next-generation operando and in-situ characterization tools. Time-resolved synchrotron X-ray absorption spectroscopy and neutron-scattering techniques sensitive to light elements will enable real-time tracking of ion dynamics under practical operating conditions. Meanwhile, cryogenic electron microscopy and advanced scanning probe microscopies will allow atomic-scale imaging and chemical mapping of interphase structures. Together, these approaches will reveal a complete and causal picture—from bulk solvation clusters to interfacial desolvation processes—thereby transforming our understanding from static inference to dynamic visualization. Such progress will fundamentally elucidate the structure–property relationships between solvation chemistry and macroscopic battery performance, ultimately accelerating the discovery of next-generation high-performance electrolytes.
3. Promising results in coin cells, which possess excess electrolyte and minimal external stress, often fail to predict performance in practical cells. Validation in large-format pouch cells under realistic conditions is the critical next step. Key parameters for assessment include: Testing under conditions such as high voltage (> 4.5 V), elevated temperature (e.g., 45–60 °C), and limited electrolyte volume to simulate realistic operating scenarios and accelerate failure mechanisms. Rigorous safety tests, including nail penetration, overcharge, and external short circuit, are imperative to evaluate the intrinsic safety advantages promised by these novel electrolytes. In-situ monitoring of gas generation during formation and cycling is essential, as certain electrolyte formulations can lead to pouch cell swelling, which compromises performance and safety.
4. *Multivalent Battery Applications* Extending these electrolyte design principles to multivalent systems (e.g., Mg^2+^, Al^3+^, Ca^2+^) presents a promising pathway for developing batteries with higher energy densities, though it requires overcoming challenges related to ion desolvation and interfacial compatibility. Designing weak-solvating or clustered solvation structures can reduce the desolvation penalty at the electrode interface, which is a major bottleneck for multivalent ion intercalation.
5. *Material Innovation and Sustainability* Future research should aim to reduce reliance on costly or fluorinated components and explore eco-friendly solvents and salts to improve sustainability and reduce overall electrolyte cost. While fluorinated compounds (e.g., LiPF₆, FEC, fluorinated ethers) often impart superior oxidative stability and facilitate robust SEI formation, they raise concerns regarding cost, environmental persistence, and potential toxicity. Research into effective non-fluorinated alternatives (e.g., phosphorus-, boron-, or sulfur-based compounds) is a priority. Electrolyte formulations should be considered with end-of-life recycling in mind. Designing electrolytes that are easier to separate and recover or that are compatible with direct recycling processes will be advantageous.
6. *Artificial Intelligence Platform for Electrolyte Design* The correlation between electrolyte formulation and battery performance remains unresolved, posing a significant challenge to the rational design of more high-performance electrolytes. Conventional strategies rely on a costly and inefficient trial-and-error process, involving iterative cycles of formulation, electrochemical testing, and optimization, which offers limited control over precise composition and solvation structure. The integration of artificial intelligence (AI) and machine learning (ML), which statistically correlate vast datasets encompassing salts, solvents [152, 153], additives, and solvation environments, can guide the prediction of novel solvent molecules, additives, formulations, and solvation architectures. This data-driven approach not only accelerates optimal electrolyte design but also enables the identification of key statistical descriptors that capture essential electrolyte properties. Consequently, it allows for the construction of predictive models that link formulation, intrinsic properties, and ultimate battery performance.

In summary, the strategic engineering of electrolyte solvation structures marks a paradigm shift in the development of next-generation batteries. While substantial progress has been made in understanding and designing these complex fluid systems, continued interdisciplinary efforts combining advanced characterization, theoretical modeling, and practical engineering will be indispensable to fully harness their potential and accelerate the transition toward more powerful, safe, and sustainable energy storage solutions.
